# Parallel Recruitment of Multiple Genes into C_4_ Photosynthesis

**DOI:** 10.1093/gbe/evt168

**Published:** 2013-10-31

**Authors:** Pascal-Antoine Christin, Susanna F. Boxall, Richard Gregory, Erika J. Edwards, James Hartwell, Colin P. Osborne

**Affiliations:** ^1^Department of Animal and Plant Sciences, University of Sheffield, United Kingdom; ^2^Department of Plant Sciences, Institute of Integrative Biology, University of Liverpool, United Kingdom; ^3^Department of Ecology and Evolutionary Biology, Brown University

**Keywords:** complex traits, co-option, evolutionary novelty, gene families, phylogenomics

## Abstract

During the diversification of living organisms, novel adaptive traits usually evolve through the co-option of preexisting genes. However, most enzymes are encoded by gene families, whose members vary in their expression and catalytic properties. Each may therefore differ in its suitability for recruitment into a novel function. In this work, we test for the presence of such a gene recruitment bias using the example of C_4_ photosynthesis, a complex trait that evolved recurrently in flowering plants as a response to atmospheric CO_2_ depletion. We combined the analysis of complete nuclear genomes and high-throughput transcriptome data for three grass species that evolved the C_4_ trait independently. For five of the seven enzymes analyzed, the same gene lineage was recruited across the independent C_4_ origins, despite the existence of multiple copies. The analysis of a closely related C_3_ grass confirmed that C_4_ expression patterns were not present in the C_3_ ancestors but were acquired during the evolutionary transition to C_4_ photosynthesis. The significant bias in gene recruitment indicates that some genes are more suitable for a novel function, probably because the mutations they accumulated brought them closer to the characteristics required for the new function.

## Introduction

The adaptation of organisms to changing environmental conditions often requires the evolution of novel traits, sometimes of impressive complexity. In many instances, the novel trait results from multiple genes, which are responsible for different morphological alterations, distinct steps in a novel biochemical cascade, or a combination of both. Genes usually do not appear de novo in a genome and the evolution of novel traits involves the co-option of preexisting genes, with alteration of their expression patterns and/or the catalytic properties of the encoded enzymes (True and Carroll 2002; Monteiro and Podlaha 2009; Tomoyasu et al. 2009). However, factors affecting the suitability of different genes for the evolution of novel traits are poorly understood.

The evolution of a given trait may require a specific enzymatic reaction, so that only genes encoding a given class of enzymes are suitable. Most enzymes are encoded by multigene families (Nei and Rooney 2005), whose members have evolved independently, in some cases for a long time. As a consequence, they have accumulated different mutations, which can affect the expression and catalytic properties of the encoded enzymes (Xu et al. 2009; Hoffmann et al. 2010; Storz et al. 2013). It could be that only certain gene lineages are suitable for a specific function during the evolution of a novel trait under the appropriate selective pressures, as suggested by the recurrent co-option of the same gene lineage for the evolution of novel adaptations (Woods et al. 2006; Zakon et al. 2006; Arnegard et al. 2010). As gene members are recurrently lost during the course of evolution (Nei and Rooney 2005), they might not be present in all species of a specific group, and their distribution might consequently affect the evolvability of a complex trait.

The diversity of evolutionary trajectories to novel traits can be investigated experimentally in a few model organisms (Weinreich et al. 2006; Blount et al. 2012; Gerstein et al. 2012). However, an experimental approach is not suitable for long-lived organisms, such as plants, where multigene families are frequent (Flagel and Wendel 2009; Guo 2013). In such instances, traits that were repeatedly acquired during evolution offer an outstanding study system (Zakon et al. 2006; Arnegard et al. 2010; Christin, Weinreich et al. 2010). C_4_ photosynthesis is one such trait that represents an excellent model system to address these questions. It consists of both morphological adaptations and the assembly of a novel biochemical cascade, which together concentrate CO_2_ before its use by the ancestral C_3_ photosynthetic apparatus, providing an advantage to plants living in a low CO_2_ atmosphere and open, warm, and dry conditions (Hatch 1987; Sage et al. 2012). Despite the involvement of multiple genes, it has evolved more than 62 times in flowering plants (Sage et al. 2011) and is especially prevalent in grasses, where it arose at least 23 times independently within the PACMAD clade (GPWGII 2012). Although genes responsible for C_4_-specific leaf anatomy, the transport of metabolites, and the cell signaling and regulation required for optimal functioning have not been precisely identified, the main enzymatic steps have long been known (Hatch and Slack 1968; Johnson and Hatch 1970; Hatch 1987; Kanai and Edwards 1999).

**Fig. 1.—:**
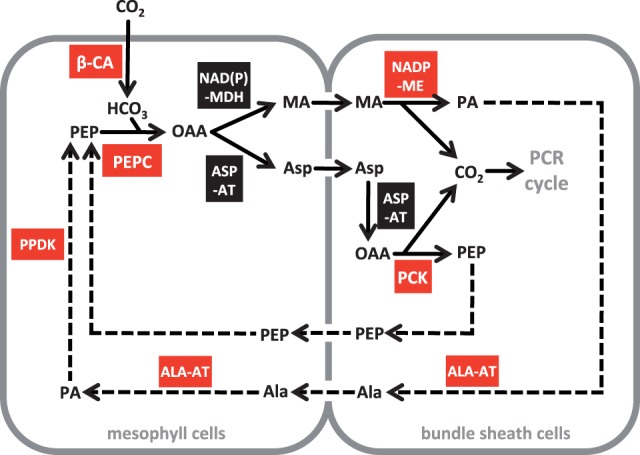
Schematic of the C_4_ cycle. Black arrows show the main reactions enabling the fixation of atmospheric CO_2_ into organic compounds in mesophyll cells until its release in bundle sheath cells, where it feeds the photosynthetic carbon reduction (PCR) cycle. Dashed arrows show the reactions allowing the regeneration of the carbon acceptors. Boxes indicate the enzymes. Those that were recruited in parallel across the three C_4_ origins are in red. Note that PCK is encoded by only a single gene lineage, which was recruited across two C_4_ origins. A more detailed schematic of the C_4_ pathway is shown in supplementary figure S1, Supplementary Material online. ALA, alanine; ALA-AT, alanine aminotransferase; ASP, aspartate; ASP-AT, aspartate aminotransferase; β-CA, β-carbonic anhydrase; MA, malate; NAD(P)-MDH, NAD(P)-malate dehydrogenase; NADP-ME, NADP-malic enzyme; OAA, oxaloacetate; PA, pyruvate; PCK, PEP carboxykinase; PCR cycle, C3 photosynthetic carbon reduction cycle; PEP, phosphoenolpyruvate; PEPC, PEP carboxylase; PPDK, pyruvate, phosphate dikinase.

In C_4_ plants, atmospheric CO_2_ is first fixed into organic acids by a combination of β-carbonic anhydrase (β-CA) and phosphoenolpyruvate carboxylase (PEPC) in leaf mesophyll cells (fig. 1 and supplementary fig. S1, Supplementary Material online). The resulting four-carbon compound is transformed and transported to bundle sheath cells (fig. 1 and supplementary fig. S1, Supplementary Material online), via various combinations of several different biochemical cascades (Kanai and Edwards 1999; Furbank 2011; Pick et al. 2011). There, CO_2_ is released by one or more of three possible decarboxylating enzymes (NAD-malic enzyme [NAD-ME], NADP-malic enzyme [NADP-ME], and phosphoenolpyruvate carboxykinase [PCK]) to feed the C_3_ photosynthetic pathway (photosynthetic carbon reduction cycle), which, in C_4_ plants, is confined to the bundle sheath cells (fig. 1). Transcript levels for all of the enzymes involved in this pathway are high during the day and are consequently easily identifiable through RNA sequencing (Brautigam et al. 2011; Gowik et al. 2011; Pick et al. 2011).

In this work, we use the convergent evolution of C_4_ photosynthesis in grasses as a model system, testing for preexisting differences in the suitability of gene family members for recruitment into a novel function within a complex biochemical pathway. Using phylogenetic analyses of whole nuclear genomes available for five grass species, we evaluate the size of C_4_-related gene families as well as the diversification of gene lineages in different subcellular compartments. We then use published and newly produced high-throughput RNA sequencing data from three grasses that evolved the C_4_ trait independently to identify and compare genes that have been independently recruited to the C_4_ pathway. The inclusion of a closely related C_3_ species for one of the C_4_ species sheds new light on the factors that might predispose particular gene lineages for a novel function.

## Materials and Methods

### Identification of Grass Gene Lineages

Phylogenetic data sets were assembled from predicted cDNA (only first transcript model for each gene) extracted from the published, complete nuclear genomes of five grasses (*Brachypodium distachyon*, *Oryza sativa*, *Sorghum bicolor*, *Setaria italica*, and *Zea mays*) as well as three distantly related eudicots with well-annotated genomes (*Populus trichocarpa*, *Arabidopsis thaliana*, and *Glycine max*). We first compiled a list of all enzymes and membrane-bound transporters with a known or putative function in C_4_ photosynthesis (Kanai and Edwards 1999; Brautigam et al. 2008; Brautigam et al. 2011). Different C_4_ subtypes are described in the older literature, which use different series of enzymes (Hatch et al. 1975; Kanai and Edwards 1999). However, accumulating evidence suggests that the classical subtypes do not represent distinct entities but can co-exist in various combinations in C_4_ plants (Shieh et al. 1982; Wingler et al. 1999; Ueno and Sentoku 2006; Muhaidat et al. 2007; Furbank 2011; Pick et al. 2011). We consequently decided to adopt a conservative approach, by considering all of the enzymes and transporters that have been associated with C_4_ photosynthesis. For each of the proteins used in the C_4_ pathway, all homologous gene sequences from *Arabidopsis* were retrieved from the GenBank database using gene annotation. *Arabidopsis* was selected as the starting point of the analyses because it has the genome with the most complete annotation of genes, especially regarding the putative function of the encoded enzymes. In addition, starting with a distant reference increased the likelihood of sampling divergent copies from grasses. The *Arabidopsis* sequences formed the initial data set and were used as the query of a Blast search based on nucleotides with a minimal *e*-value of 0.00001 against one of the published complete nuclear genomes. Positive matches were retrieved and added to the data set, which was then used as the query for a Blast search against the next genome. This process was iterated until all complete genomes (including *Arabidopsis*) had been successively screened.

Each final data set was translated into amino acids and aligned using ClustalW (Thompson et al. 1994). The alignment was manually inspected, and sequences that corresponded to partial cDNA or that were clearly not homologous to the *Arabidopsis* reference (false positives) were removed. A gene family phylogenetic tree was then inferred from the recovered nucleotide sequences under maximum likelihood, as implemented in PhyML (Guindon and Gascuel 2003), under a general time reversible (GTR) substitution model with a gamma shape parameter. Statistical support was evaluated with 100 bootstraps. The resulting phylogenetic tree was manually inspected and groups of orthologous genes were identified as well-supported clades of grass genes, for which relationships were compatible with the species relationships based on other markers (GPWGII 2012).

For each predicted cDNA extracted from complete genomes, the presence of a putative chloroplast transit peptide, directing the pre-protein to the chloroplast, was tested using the chloroP prediction software 1.1 (Emanuelsson et al. 1999).

### Sampling Design

**Fig. 2.—:**
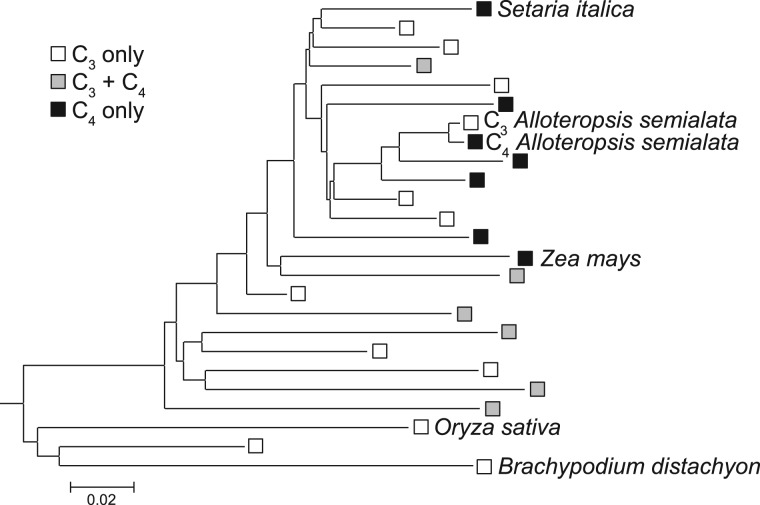
Simplified phylogeny of grasses showing the relationships between the sampled taxa. The phylogenetic tree was retrieved from Grass Phylogeny Working Group II (2012). Subfamilies are compressed, with the exception of the Panicoideae containing *Zea*, *Setaria*, and *Alloteropsis*, for which groups not containing these taxa are compressed. The photosynthetic types of taxa present in each group are indicated near the tip; white = all C_3_, black = all C_4_, gray = both C_3_ and C_4_.

High-throughput RNA sequencing data has been published for leaves of two C_4_ grass species for which a complete nuclear genome is available, *S. italica* and *Z. mays* (Li et al. 2010; Bennetzen et al. 2012). Both species belong to the same grass subfamily (Panicoideae) but evolved C_4_ photosynthesis independently (GPWGII 2012; fig. 2). A third C_4_ taxon, namely *Alloteropsis semialata* subsp. s*emialata*, for which there were no existing genomic or transcriptomic data sets, was also included in the analysis. This taxon also belongs to Panicoideae but represents an additional C_4_ origin in this hotspot of C_4_ evolution (Christin et al. 2012; GPWGII 2012; fig. 2). These three species use the C_4_ biochemical pathway based on the decarboxylating enzyme NADP-ME (Gutierrez et al. 1974; Ueno and Sentoku 2006). In the case of *Zea* and *Alloteropsis*, this pathway is complemented by a shuttle based on the enzyme PCK, which in the latter can represent the majority of carbon flux through the C_4_ pathway (Prendergast et al. 1987; Wingler et al. 1999; Ueno and Sentoku 2006; Pick et al. 2011).

In addition to these three C_4_ taxa, the C_3_ taxon *Alloteropsis semialata* subsp. *eckloniana* was analyzed. This taxon is closely related to the C_4_ *Alloteropsis*, with a divergence time estimated at ∼3 Ma (Ibrahim et al. 2009; Christin et al. 2012; fig. 2). The transcriptomes of the C_3_ and C_4_ *Alloteropsis* have been analyzed previously for a different purpose (Christin et al. 2012), but additional data were produced for this study.

### Sequencing and Assembly of *Alloteropsis* Transcriptomes

Seeds of C_4_ *Alloteropsis semialata* (R.Br.) Hitchc. subsp. *semialata* and C_3_ *Alloteropsis semialata* (R.Br.) Hitchc. subsp. *eckloniana* (Nees) Gibbs Russell were collected from plants that had been open pollinated in South Africa. Seeds were obtained from a wild population of the C_3_ *Alloteropsis* growing near Grahamstown (Port Elizabeth, Eastern Cape), and from a common garden population of the C_4_ *Alloteropsis* growing in the same area, but originally collected from a wild population near Middelburg (Pretoria, Mpumalanga).

Seeds were germinated under sterile conditions on 1.2% plant agar containing 50 mg/l gibberellic acid in order to achieve rapid and uniform germination. Plants were grown in 600 ml pots containing a 1:1 mix of M3 compost:perlite designed to provide a free-draining, high nutrient medium (LBS Horticulture, Colne, Lancs, UK) and placed within a climate controlled plant growth cabinet (Fitotron PG660, Gallenkamp, Loughborough, UK) under a 16:8 h day:night cycle, a mean daytime photon flux density of 550 µmol m^−2 ^s^−1^, day:night temperatures of 25:20 °C, and 70% humidity. Plants were watered twice weekly and fertilized using Long Ashton solution at increasing strength and frequency as the plants grew larger (to a maximum of full-strength solution applied weekly). Plants were raised under these conditions for 8 weeks, before the day:night cycle was changed to 12:12 h for a further 5 weeks prior to sampling. The youngest fully expanded leaf was sampled from randomized biological quadruplicates every 4 h over the 12:12 h light:dark cycle starting immediately after the lights came on at “dawn,” snap-freezing samples in liquid nitrogen and storing them at −80 °C until processing them for total RNA isolation. Each replicate at each time point was taken from a different plant, so that a total of 24 plants of each subspecies were sampled over the diurnal cycle.

Frozen leaf samples were ground in liquid nitrogen using a mortar and pestle. Total RNA was isolated from the frozen ground leaf tissue using the Qiagen RNeasy kit following the manufacturer’s protocol but using 450 µl of the kit’s RLC extraction buffer modified with the addition of 4.5 µl β-mercaptoethanol and 13.5 µl 50 mg/ml polyethylene glycol 20,000 per sample. Part of the RNA was saved for semi-quantitative polymerase chain reaction (discussed later). Prior to the generation of full-length double-stranded cDNA for 454 library production, the rest of the total RNAs were pooled in equimolar amounts giving equal weight to each sampling point to generate four pools of *Alloteropsis* total RNA, namely C_3_ dark, C_3_ light, C_4_ dark, and C_4_ light. After thorough mixing, each pool of total RNA was used for oligo-dT primed synthesis of full-length double-stranded cDNA using the SMARTer cDNA synthesis kit (Clontech, Mountain View, CA). Each sample of full-length cDNA was then used for Roche 454 sequencing library production using the manufacturer’s recommended procedures. Each library was initially sequenced on a quarter of a Titanium plate using the Roche 454 GS-FLX sequencer (table 1). Extra sequencing was performed for the C_4_ samples in order to achieve superior assemblies and to compensate for the poor initial run of the C_4_ dark sample, which had only produced 32,874 reads (see table 1).

**Table 1:** Statistics for *Alloteropsis semialata* 454 Runs

RNA Sample/454 Library	454 Plate Scale	Reads	Total bp Per Run	Total Combined bp Per RNA Sample	Average Read Length (bp)
C_3_ light	Quarter plate	175,706	46,838,328	46,838,328	267
C_3_ dark	Quarter plate	141,516	32,326,469	32,326,469	228
C_4_ light	Quarter plate	179,678	38,885,333	92,876,291	260
C_4_ dark	Quarter plate	32,874	2,790,964	83,333,222	289
C_4_ light	Quarter plate	177,932	53,990,958		
C_4_ dark	Quarter plate	209,295	68,844,227		
C_4_ dark	Eighth plate	46,030	11,698,031		

De novo transcriptome assemblies based on the 454 data were undertaken separately for the C_3_ and C_4_ *Alloteropsis*. The reads produced by the 454 Titanium sequencing were each trimmed for poly-A/T tails and 454 and SMARTer adapter sequences (in-house tool, based on a multi-pass Blast and heuristics), with reads trimmed to less than 50 bp removed. Trimming reduced the number of C_3_ reads to 253,682 (68,253,971 bp) and the number of C_4_ reads to 538,682 (155,267,063 bp). The trimmed C_3_ and C_4_ reads were then assembled with MIRA (Chevreux et al. 2004) using the default parameters implied by the settings “–job=denovo,est,accurate.” The resulting C_3_ and C_4_ assemblies produced 15,892 contigs (7,375,929 bp) and 39,549 contigs (22,259,361 bp), using 191,136 and 400,726 reads, respectively, with N50 (>200 bp) of 504 and 449 bp.

Reads per contig were counted using the .ace file produced by the assemblies and then normalized to reads per kilobase of contig length per million reads (rpkm) values to account for the variation in number of C_3_ and C_4_ reads. The contigs were then mapped to the *Arabidopsis* peptide reference (TAIR10, http://www.arabidopsis.org/, last accessed November 4, 2013) using Blast (*E* = 1^−5^).

### Diurnal Regulation of the Transcript Abundance of C_4_-Related Genes in *Alloteropsis*

Putative C_4_-specific contigs of *Alloteropsis* were identified in silico as contigs with a higher 454 read abundance in the C_4_ sample relative to the C_3_ sample. Of these, contigs with a differential transcript abundance between the light and dark reads in the C_4_ subspecies were selected for more detailed analysis with semi-quantitative RT-polymerase chain reaction. cDNA was synthesized from the total RNA extracted from different individuals at different times using the Qiagen Quantitect RT kit which uses an optimized blend of oligo-dT and random primers to promote high cDNA yields, even from 5′ regions. The Quantitect RT kit also includes a genomic DNA wipeout buffer for the removal of contaminating genomic DNA from total RNA prior to reverse transcription. The resulting cDNA was diluted 1:5 with molecular biology grade water prior to use for semi-quantitative PCR.

**Fig. 3.—:**
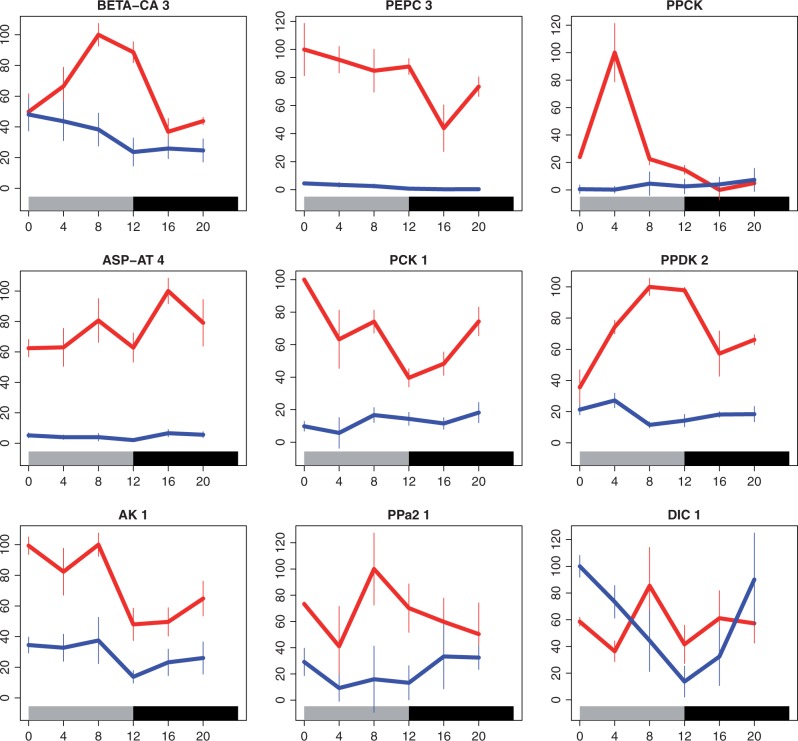
Diurnal regulation of enzymes of the C_4_ pathway. For six enzymes and three putative metabolite transporters of the C_4_ pathway, the normalized transcript abundance is indicated over the course of a day, with time shown in hours after dawn. Values are comparable within each panel but not among panels. For each sample point, standard errors were calculated from three replicates. Values measured in the C_3_ *Alloteropsis* are in blue and those measured in the C_4_ *Alloteropsis* are in red. The gray bar at the bottom represents the light period and the black bar the dark period.

Polymerase chain reactions were performed using 1 µl of each cDNA sample in a reaction mixture (10 µl) containing 1× Sigma REDTaq ReadyMix PCR reaction mix with MgCl_2_ (Boxall et al. 2005). The gene-specific primers used to amplify each gene are listed in supplementary table S1, Supplementary Material online. All primers produced amplification products of the expected size based on the corresponding 454 contig sequence. All PCR products were separated on 1% agarose gels in 1× Tris-acetate EDTA and stained in ethidium bromide. Gels were visualized using a Geneflash gel documentation system and images captured electronically onto a memory card. PCR product band intensities were quantified using Metamorph software. A polyubiquitin gene orthologous to the *Arabidopsis* polyubiquitin UBQ10 gene (AT4G05320) was used as a reference gene for the PCR analysis. The quantified PCR signals for each C_4_ gene were normalized to the UBQ10 signal to correct for minor variations in the loading of RNA into the RT reactions and/or the efficiency of the RT reactions. PCRs were performed on biological replicates, and the quantitative data shown in the figure 3 and supplementary figure S2, Supplementary Material online, represent the mean of three biological reps.

### Phylogenetic Annotation of *Alloteropsis* Contigs

In order to accurately assign the assembled transcripts of the C_3_ and C_4_ *Alloteropsis* to groups of orthologs, each contig homologous to any C_4_-related gene was successively placed in a phylogeny with the corresponding reference data set extracted from complete nuclear genomes. For each C_4_ enzyme, the reference data set was used as a query in a Blast search against the C_3_ and the C_4_ assembled transcriptomes based on nucleotides with a maximal *e*-value of 0.001. For each positive match, the longest matching sequence was extracted from the Blast result. The *Alloteropsis* nucleotide sequence was aligned with the reference data set using MUSCLE (Edgar 2004), and a phylogenetic tree was inferred using PhyML and a GTR model. The resulting phylogenetic tree was inspected visually and the *Alloteropsis* contig was assigned to one of the gene lineages defined based on complete genomes when unambiguously nested in the clade. In some cases, contigs were not assignable to any gene lineage because they were too short or poorly aligned and were consequently positioned outside the groups of orthologs based on complete genomes. This problem concerned only a small number of contigs associated with small rpkm values. These were discarded. The relative transcript abundance for each gene lineage was then assessed by summing the 454 rpkm of all the contigs assigned to this lineage.

The phylogenetic annotation of contigs was not feasible for some C_4_-related families of genes, which are composed of a large number of closely related genes, hampering a confident identification of gene lineages generated by ancient gene duplications. Of the known enzymes of the C_4_ pathway, only phosphoenolpyruvate carboxylase kinase (PPCK) was not analyzed phylogenetically because of a large number of related genes. The phylogenetic annotation was also not applied to several candidate transcription factors for the same reason (discussed later).

### Estimations of Transcript Abundance for Genes from *Setaria* and *Zea*

Data from 11 Illumina runs reported for *S. italica* in a previous study (Bennetzen et al. 2012) were retrieved from the NCBI database. These include multiple replicates taken at 3 h into the light (of a 12 h light cycle), at four different positions along the leaf corresponding to different stages of a developmental gradient (Li et al. 2010; Bennetzen et al. 2012). The paired-end Illumina reads from each run were successively mapped on *Setaria* predicted cDNA using Bowtie2 (Langmead and Salzberg 2012). A mixed model was used, which allowed unpaired alignments when paired alignments were not possible. Only one best alignment was reported per read. The transcript abundance for each predicted cDNA was estimated as the number of times the cDNA was the reported match. After correcting for the total number of mappable reads (in millions) and the length of the predicted cDNA (in kilobases), this produced rpkm values for each predicted cDNA. When multiple predicted cDNAs were assigned to the same gene lineage, the rpkm values were summed. Values were averaged among biological replicates.

The same procedure was used to estimate the transcript abundance of each gene lineage in *Z. mays*. Two replicates were previously sequenced along a similar development gradient, for each of the four developmental stages (Li et al. 2010). These were sequenced as single-end Illumina reads and were consequently mapped as such against *Zea* predicted cDNA using Bowtie2.

### Identification of Gene Lineages Recruited in Each C_4_ Origin

For each gene family, the groups of orthologs containing the putative C_4_-specific gene was identified as the gene lineage with a transcript abundance greater than 300 rpkm in the day sample for the C_4_ *Alloteropsis* and in each of the C and D segments in *Setaria* and *Zea*. For *Alloteropsis*, the C_4_ specificity was confirmed by a higher transcript abundance in the C_4_ than in the C_3_ *Alloteropsis* and a higher abundance in the C_4_ during the light than in the dark. For *Zea* and *Setaria*, the C_4_ specificity was confirmed by an increase in transcript levels during the development of mature leaves (such that the average of segments C and D was greater than the average of segments A and B).

### Statistical Test for Randomness of Gene Recruitment

A total of 100,000 replicates were obtained by sampling three times with replacement gene lineages from vectors corresponding to the number of identified gene lineages in each gene family. For each replicate, the number of enzymes for which the same gene lineage was recruited in all species was recorded. The distribution of the simulated number of convergent recruitments was used to obtain the probability of obtaining by chance a value equal to or larger than the observed value.

### Comparison of Closely Related Duplicated Genes

For each identified C_4_-specific gene lineage of *Setaria* and *Zea*, the presence of duplicated genes was inferred when multiple, nonidentical, genes were assigned to the same lineage. This approach was not applicable to *Alloteropsis*, because the incompleteness of most contigs due to the limited size of the 454 transcriptome data set prevented pairwise comparisons. For each group of identified C_4_-related recent duplicates, the expression level of each gene was retrieved. The values for two β-CA genes from *Setaria* were averaged because these duplicates did not differ in their coding sequence. The approach might be partially biased because closely related duplicates could be insufficiently different to confidently assign reads, and reads of one of the duplicates might occasionally be mapped to the other gene. However, the analysis should still detect differential expression between recent duplicates.

### Identification of Potential C_4_-Related Transcription Factors

In addition to genes encoding enzymes of the C_4_ biochemical pathway, the comparison of transcript abundance in C_3_ and C_4_ *Alloteropsis* identified seven transcription factors that are more highly expressed in the C_4_ plants. Semi-quantitative polymerase chain reaction confirmed that the transcript abundance of these genes in the C_4_ tissues varied diurnally and peaked during the light phase (supplementary fig. S2, Supplementary Material online). The peak was consistently higher in the C_4_ *Alloteropsis* compared with the C_3_. The highest difference was found for BIN4, which is transcribed at a relatively high level in the C_4_ leaves but was not detected in the C_3_ transcriptome (supplementary table S2 and fig. S2, Supplementary Material online). These transcription factors represent candidates for a role in the C_4_-specific regulation.

Four of the identified transcription factors belong to large gene families with a large number of members, with homology sometimes limited to only certain parts of the sequence. The size of these gene families prevented phylogenetic analyses, which we limited to three genes. The transcription levels of these three candidates along the developmental gradient in *Setaria* and *Zea* did not suggest C_4_ function (supplementary table S2, Supplementary Material online), which might mean either that these genes have a C_4_-related function only in *Alloteropsis* or that the detected diurnal upregulation in the C_4_ *Alloteropsis* is not linked to C_4_ photosynthesis.

## Results

### Phylogenetics of Gene Families and Subcellular Localization

We first obtained a well-resolved phylogenetic tree for each gene family, using the genomes of five grasses and three eudicots, for which complete or draft sequences are available (supplementary figs. S3 and S4, Supplementary Material online). In each case, it was possible to delimit groups of orthologous genes for the grass genomes. Between one (for PCK) and seven (for PEPC) gene lineages were identified.

For most gene families, some of the genes were predicted to be chloroplast-specific, and many of the gene lineages included mixtures of genes with and without chloroplast transit peptides (supplementary figs. S3 and S4, Supplementary Material online). In most cases, the subcellular localization predicted on the basis of sequences corresponded to the subcellular localization reported in the literature. Exceptions included the putative C_4_ aspartate aminotransferase (ASP-AT) of *Setaria* and the putative C_4_ alanine aminotransferase (ALA-AT) of *Setaria*, *Zea*, and *Alloteropsis*, which were predicted to be chloroplast targeted, whereas the enzyme is reported in some literature to act in the cytosol or the mitochondria of C_4_ plants (e.g., Kanai and Edwards 1999; Furbank 2011). However, a localization of ASP-AT in the chloroplasts was proposed by earlier authors (e.g., Ku et al. 1981; Shieh et al. 1982) and supported recently for maize by transcriptomics (Pick et al. 2011; Chang et al. 2012). In addition, one of two *Arabidopsis* genes encoding PCK is predicted to be chloroplast targeted, although both genes have previously been assumed to encode cytosolic forms (Malone et al. 2007). This discrepancy might result from errors in the prediction of transit peptides or in the gene models. Alternatively, the prediction might represent a real biological phenomenon. For instance, some genes might encode both cytosolic and chloroplast forms through different promoters, as is the case for some genes encoding PPDK (supplementary fig. S3, Supplementary Material online; Sheen 1991; Parsley and Hibberd 2006).

### Identification of C_4_ Forms and Convergent Recruitment

For most of the major C_4_ enzymes, one of the genes was more abundantly transcribed in the C_4_ accession of *Alloteropsis* during the day than the others (supplementary table S2, Supplementary Material online). This gene was expressed at low levels in the C_3_ accession, with the exception of one gene encoding β-CA, which was highly expressed in the C_3_ but at comparatively lower levels than in the C_4_ (supplementary table S2, Supplementary Material online). In addition, the same gene was more abundant in the C_4_ *Alloteropsis* during the light phase than during the dark, again with the exception of one gene encoding β-CA, which was present at extremely high abundance in both the light and dark periods. C_4_-specific genes of *Alloteropsis* were identified for a total of eight enzymes (table 2). A second gene encoding ASP-AT had a rpkm value above 300, but its transcript abundance was similar to the C_3_ *Alloteropsis* and 20 times lower than another gene encoding ASP-AT (supplementary table S2, Supplementary Material online). One of the gene lineages for AK and PPa was present at higher transcript abundance in the C_4_ *Alloteropsis* during the day, but the transcript abundance of these genes also increased from night to day in the C_3_ *Alloteropsis*. None of the metabolite transporters expected to be required for the C_4_ system to function efficiently in *Alloteropsis* had high transcript abundance in the C_4_ compared with the C_3_ *Alloteropsis*, preventing the identification of C_4_-specific genes for these important steps in the hypothesized pathway.

**Table 2:** Summary of C_4_ Enzyme Recruitment

Enzyme	Total Number of Gene Lineages	Number of C_4_ Recruitment Events Identified	Convergent Recruitment
ALA-AT	5	3	Yes
ASP-AT	4	3	No
β-CA	3	3	Yes
NAD(P)-MDH	4	3	No
NADP-ME	4	3	Yes
PCK	1	2	NA
PEPC	7	3	Yes
PPDK	2	3	Yes

**Fig. 4.—:**
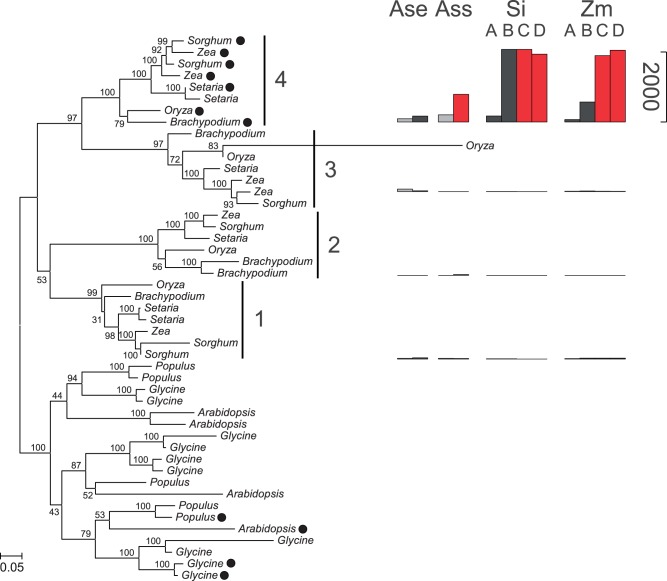
Multigene family encoding NADP-ME. Bootstrap values are indicated near branches. See supplementary figure S3, Supplementary Material online, for gene accession numbers. Gene lineages are delimited on the right. Black circles indicate predicted chloroplastic targeting. For each gene lineage, barplots on the right are proportional to the rpkm value in different species (Ass = C_4_ *Alloteropsis*; Ase = C_3_ *Alloteropsis*; Si = *Setaria*; Zm = *Zea*), different conditions for *Alloteropsis* (black = day; gray = night), and different stages of development for *Setaria* and *Zea* (A = base of the leaf; B = transitional; C = maturing; D = mature). Abundances of putative C_4_-specific forms are in red. For this enzyme, the three C_4_ origins recruited the same gene lineage number 4.

**Fig. 5.—:**
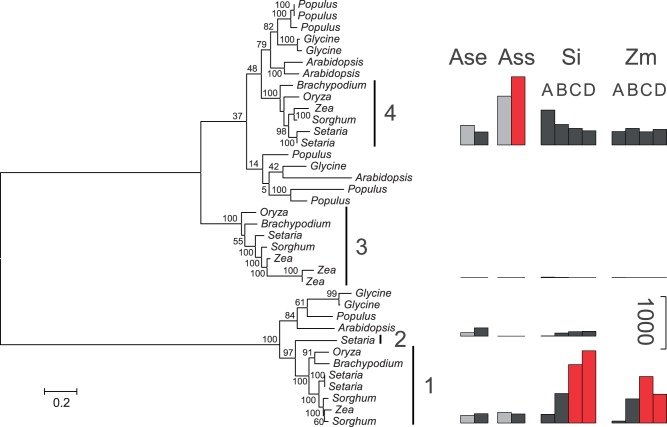
Multigene family encoding NAD(P)-MDH. Bootstrap values are indicated near branches. See supplementary figure S3, Supplementary Material online, for gene accession numbers. Gene lineages are delimited on the right. For each gene lineage, barplots on the right are proportional to the rpkm value in different species (Ass = C_4_ *Alloteropsis*; Ase = C_3_ *Alloteropsis*; Si = *Setaria*; Zm = *Zea*), different conditions for *Alloteropsis* (black = day; gray = night), and different stages of development for *Setaria* and *Zea* (A = base of the leaf; B = transitional; C = maturing; D = mature). Abundances of putative C_4_-specific forms are in red. For this enzyme, the C_4_ ancestors of *Setaria* and *Zea* recruited the gene lineage 1 while the C_4_ *Alloteropsis* recruited the gene lineage 4.

The C_4_-specific genes from *Zea* were identified for the same eight enzymes (table 2). PCK is encoded by a single gene lineage and was excluded from analyses. Of the remaining seven cases, five of the C_4_-specific genes from *Alloteropsis* and *Zea* belonged to the same gene lineages (table 2 and fig. 4). Exceptions were ASP-AT and NAD(P)-MDH (table 2 and fig. 5). Different members of the NAD(P)-MDH gene family encode either NADP-dependent MDH (NADP-MDH) or NAD-dependent MDH (NAD-MDH). Previous work has shown that *Zea* uses NADP-MDH for its C_4_ pathway (Sheen and Bogorad 1987), whereas *Alloteropsis* uses NAD-MDH (Ueno and Sentoku 2006), and this explains why the two taxa recruited different genes, one of which (NAD-MDH) is predicted to be cytosolic and the other (NADP-MDH) chloroplastic (supplementary fig. S3, Supplementary Material online).

Finally, for six enzymes, the C_4_-specific genes used by *Setaria* were unambiguously identified, but for ASP-AT two different genes were present at high transcript abundance in the leaves*.* One of them corresponded to the most highly expressed gene lineage in the C_4_ *Alloteropsis* and the other to the most highly expressed gene lineage in *Zea*. Multiple forms of this enzyme might be used for C_4_ photosynthesis, as proposed for other species (Taniguchi and Sugiyama 1990; Chang et al. 2012) or have one copy expressed at high levels for a different reason. As *Alloteropsis* and *Zea* use different gene lineages for this enzyme, the ambiguity in *Setaria* does not affect the tally of convergent recruitment (table 2). Finally, none of the genes encoding PCK were expressed at high levels in the leaves of *Setaria* (table 2), which is consistent with the hypothetical C_4_ “NADP-ME subtype” pathway of this species that does not involve this decarboxylating enzyme (Gutierrez et al. 1974; Kanai and Edwards 1999).

The same AK and PPa gene lineages increased in abundance along the developmental gradient in *Zea* and *Setaria* and were present at high transcript abundance in the C_4_ *Alloteropsis*, but these were also present at high transcript abundance in the C_3_ *Alloteropsis*. Several metabolite transporters showed an increase of transcription along the developmental gradient in *Zea* and *Setaria* (supplementary table S2, Supplementary Material online). However, these were not considered in the analysis of convergent recruitment because no C_4_-specific transporter could be identified from *Alloteropsis* transcriptomes. Our estimate of parallel gene recruitment is therefore conservative.

**Fig. 6.—:**
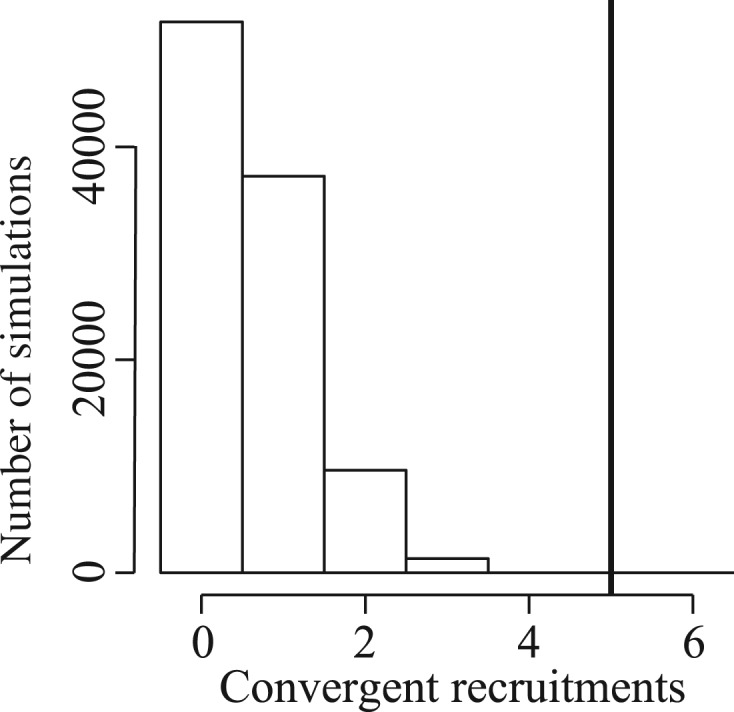
Simulated distribution of the number of convergent recruitment events. The observed value is indicated by the black vertical bar.

In total, excluding PCK, the C_4_-specific gene lineages were identified for seven enzymes that are common to the C_4_ pathway of all three species (table 2). For five of these, the same gene lineage was independently recruited in each of the three C_4_ origins (e.g., fig. 4). Given the size of the gene families, five cases of convergent recruitment are highly significantly greater than expected by chance (*P* <0.00005; fig. 6). This provides strong evidence for a bias in the recruitment of genes for a C_4_-specific function.

### Differential Expression of Closely Related Duplicated Genes

For *Setaria*, duplicates within C_4_-specific gene lineages were identified only for β-CA. In this case, only one of the duplicates showed a very strong pattern of development-dependent transcript abundance (table 3). The other duplicate was only detected at low levels along the developmental gradient.

**Table 3:** Expression of Closely Related Gene Duplicates

Enzyme	Lineage	Gene	A^a^	B^a^	C^a^	D^a^
β-CA	3	Si003882	80	2,157	6,769	8,297
β-CA	3	Si002140/Si002148	2	22	15	6
β-CA	3	GRMZM2G121878	3	573	2,868	1,782
β-CA	3	GRMZM2G348512	6	300	1,308	792
β-CA	3	GRMZM2G094165	4	192	602	334
NADP-ME	4	GRMZM2G085019	5	501	1,810	1,963
NADP-ME	4	GRMZM2G122479	63	68	86	84
PCK	1	GRMZM2G001696	1	11	741	1,408
PCK	1	GRMZM5G870932	4	2	70	146
PPDK	2	GRMZM2G306345	64	6,106	18,544	16,386
PPDK	2	GRMZM2G097457	1	94	396	356

^a^Expression level (in rpkm) along four stages of a leaf developmental gradient, from A to D, which is the most mature stage.

Duplicates were found within five C_4_-specific gene lineages of *Zea* (table 3). In each case, one of the duplicates was expressed at very high levels compared with the others. The other duplicate of NADP-ME showed a constant expression level along the developmental gradient, but for the four other enzymes, the second duplicate also showed an increase of expression along the developmental gradient. Whether the increase of expression of the second duplicate is real or results from the erroneous mapping of some reads is out of reach of the present data set.

### Diurnal Regulation of C_4_-Related Enzymes

In the C_4_ *Alloteropsis*, the steady-state transcript abundance of a number of C_4_-related genes oscillated over the light–dark cycle (fig. 3). In particular, transcript levels of several core C_4_ genes, including CA, PEPC, PPDK, PCK, and plastidic adenylate kinase (AK), displayed a broad transcript peak in phase with the light period. PEPC and CA reached minimum levels in the first half of the dark period. For CA, nocturnal transcript levels were similar for both the C_3_ and C_4_ *Alloteropsis*, implying that the difference between them associated with C_4_ photosynthesis is an increased transcript level during the day. PPDK transcript levels displayed a broad light-period phase peak declining to a trough at the end of the dark period. PCK transcript levels peaked at dawn, were high for the first half of the light period, reached their minimum at dusk, and subsequently increased gradually through the dark period. Light–dark oscillations in the transcript abundance of the orthologous genes in the C_3_ *Alloteropsis* were either negligible (PEPC, ASP-AT) or very small in amplitude relative to those of the C_4_ subspecies (PCK, CA, PPDK, plastidic AK).

## Discussion

### Biased Recruitment Indicates Differences in C_4_ Suitability among Genes

The phylogenomic analysis of sequences produced by high-throughput sequencing methods indicates that the recruitment of genes for the C_4_ pathway was not random (table 2 and fig. 6). The three species considered in this study belong to the grass subfamily Panicoideae, but they are members of different C_4_ lineages, which are separated in the phylogeny by numerous C_3_ lineages and shared a last common ancestor more than 25 Ma (Christin et al. 2008; Vicentini et al. 2008; GPWGII 2012; fig. 2). Multiple lines of evidence, including comparative analyses of foliar anatomies and C_4_ genes, support multiple C_4_ origins over an ancestrally C_4_ type with multiple losses in the C_3_ lineages (Christin, Freckleton et al. 2010). It is striking that these three independent evolutionary transitions from C_3_ to C_4_ photosynthetic types recruited the necessary enzymes from the same ancestral gene lineages.

All enzymes of the C_4_ pathway already existed in the C_3_ ancestors, but they were responsible for different, generally non-photosynthetic, functions (Monson 2003; Aubry et al. 2011). Their enzymatic reaction is, however, conserved between C_3_ and C_4_ plants and, theoretically, any of the different forms might have been recruited into a C_4_ function. This is disproved by the recurrent use of the same gene lineage out of several available in grass genomes, which indicates that certain ancestral genes are predisposed to take on the C_4_-specific function. The suitability of a particular gene for a new function depends on a number of factors, including the suitability of expression patterns and catalytic properties of the encoded enzyme for its new function, or the capacity to quickly acquire the required properties through a few key mutations (Christin, Weinreich et al. 2010). In addition, the availability of a gene can depend on its ancestral function, which might prevent neofunctionalization if the fitness cost of losing the ancestral function outweighs the fitness benefit of its new function.

### Suitability of Expression Patterns

Different forms of some C_4_-related enzymes have different expression patterns, in terms of diurnal regulation and the tissues, cells, and subcellular compartments in which the enzyme is expressed (Maurino et al. 1997; Finnegan et al. 1999; Tausta et al. 2002; Alvarez et al. 2013). A function in C_4_ photosynthesis requires light-induced high expression levels in specific cell types of the leaf (Sheen 1999). The C_3_ *Alloteropsis* is the first C_3_ member of the PACMAD clade, the group that encompasses all C_4_ grasses together with numerous C_3_ taxa (GPWGII 2012), which has had its transcriptome analyzed at this level of detail. None of the C_4_-related genes shows diurnal variation in C_3_ *Alloteropsis* similar to that observed in the C_4_ *Alloteropsis* (fig. 3 and supplementary table S2, Supplementary Material online), indicating that the C_4_-specific diurnal cycle did not predate the evolution of C_4_ photosynthesis but was acquired during the transition from C_3_ to C_4_ photosynthesis.

It is noteworthy that, for most C_4_-related enzymes, the most abundantly transcribed gene lineage in the mature leaves of C_3_ *Alloteropsis* is the one that has been recruited in the C_4_ pathway (supplementary table S2, Supplementary Material online). This observation is consistent with the hypothesis that an ancestrally higher transcript level in leaves increased the likelihood of these genes becoming C_4_-specific. The evolution of C_4_-specific forms then occurred through a strong increase of these leaf expression levels, together with the strengthening of the diurnal cycle and often an altered phasing of the daily transcript peak relative to dawn and dusk. The gene encoding β-CA that is orthologous to the C_4_-specific forms was detected at especially high transcript levels in leaves of the C_3_ *Alloteropsis* (fig. 3 and supplementary table S2, Supplementary Material online). High transcript abundance of specific β-CA lineages has been observed in other C_3_ taxa, where it optimizes the relative concentration of CO_2_ for Rubisco (Badger and Price 1994; Ludwig 2011) and probably predisposed these β-CA lineages for a C_4_ function, which evolved through a further increase in daytime transcript levels.

The suitability of ancestral genes for a C_4_ function might also depend on their subcellular localization. The integrity of the C_4_ cascade requires some enzymes to work in the cytosol while others must be active in particular organelles. In the case of NADP-ME, chloroplast-targeting evolved only once, at the base of one of the gene lineages (“grasses 4,” fig. 4 and supplementary fig. S3, Supplementary Material online), which was then recruited to the C_4_ pathway at least six times independently (fig. 4; Maurino et al. 1997; Christin, Samaritani et al. 2009). NADP-ME uses NADP^+^ as a co-factor, which is abundantly produced in the chloroplasts. The abundance of the co-factor as well as the vicinity of CO_2_ release to Rubisco activity might predispose chloroplastic forms of NADP-ME for a function in the C_4_ pathway, explaining the observed recruitment bias (table 2; Christin, Samaritani et al. 2009). The presence or absence of chloroplast transit peptides might similarly have excluded some other genes from a C_4_ function, but this mechanism alone is insufficient to explain the observed convergent pattern, because multiple genes with the required subcellular expression for C_4_ photosynthesis exist in all other gene families (supplementary fig. S3, Supplementary Material online). However, it should be noted that our analysis does not consider the expression in other subcellular compartments (e.g., mitochondria), because these are more difficult to predict with accuracy from sequence data alone.

Finally, the suitability of genes for C_4_ photosynthesis might be determined by their cell specificity. The expression of most enzymes in either the mesophyll or the bundle sheath cells of leaves is instrumental for the C_4_ pump (fig. 1; Sheen 1999; Hibberd and Covshoff 2010). The expression analyses presented in this study are not able to differentiate these two tissues, but other techniques can identify cell-specific expression. Elements determining bundle sheath-specific expression are already present in some C_4_-related genes of C_3_ plants (Brown et al. 2011; Kajala et al. 2012), but their distribution among gene lineages is unknown. Depending on the distribution of cell-specific regulatory motifs among gene lineages, these could represent a key determinant of C_4_ suitability.

### C_4_ Suitability as a Function of Catalytic Properties and Gene Availability

In addition to expression patterns, the enzymes encoded by different members of the same gene family also differ in their catalytic properties (Ting and Osmond 1973; Tausta et al. 2002; Svensson et al. 2003; Alvarez et al. 2013). In several of the C_4_-related genes, the evolution of C_4_-specific forms involved adaptive mutations of the coding sequence, which suggests catalytic modifications during the C_3_ to C_4_ transition (PEPC [Christin et al. 2007; Wang et al. 2009]; PCK [Christin, Petitpierre et al. 2009]; NADP-ME [Christin, Samaritani et al. 2009, Wang et al. 2009]; CA, PPDK, PPDK-RP [Wang et al. 2009]). The different members of gene families might have possessed catalytic properties that made them differentially distant from the C_4_ requirements, influencing their suitability for a C_4_ function. Unfortunately, previous biochemical studies have generally characterized only a subset of the isoforms encoded by a given gene family, whereas a comparison of the catalytic properties of all gene lineages identified in the genome of a given species would be required to understand the impact of catalytic properties on C_4_ suitability.

Finally, the C_4_ suitability of genes has often been hypothesized to depend on their redundancy, with gene duplications removing the functional constraints on gene diversification (Monson 2003; Sage 2004). This hypothesis is difficult to evaluate rigorously. Gene duplications linked to C_4_-specific genes were detected for half of C_4_-related enzymes in the polyploid *Zea* (table 3) but for only one in the diploid *Setaria*. In all cases, only one of the duplicates was expressed at very high levels, suggesting that the gene duplication preceded the modification of expression levels. Previous phylogenetic studies have shown that, in several instances, the evolution of C_4_-specific genes for PCK quickly followed a gene duplication (Christin, Petitpierre et al. 2009). However, the importance of gene duplication was not apparent in the origin of other C_4_ enzymes, such as PEPC, where one paralog was recruited in the absence of gene duplication other than those predating the diversification of grasses tens of millions of years earlier (Christin et al. 2007). The requirement for gene duplication might depend on the size of the gene families as well as the functional similarity between different gene lineages. PCK is the only C_4_-related enzyme present as a single gene lineage in grass genomes (supplementary fig. S3, Supplementary Material online) and, strikingly, it is also the enzyme for which the highest number of C_4_ origins were preceded by a gene duplication (Christin, Petitpierre et al. 2009). A complete understanding of the functional diversity present in the different gene families would however require the functional characterization of each gene lineage, because orthology is a poor predictor of functional similarity (Studer and Robinson-Rechavi 2009).

## Conclusions

Using phylogenetic analyses to compare the transcriptomes of one C_3_ and three independently evolved C_4_ grasses, we showed that the same members of five gene families have been recurrently recruited for a function in the C_4_ pathway. This unexpected result implies that some members of gene families are more suitable than others for the evolution of novel adaptations. The properties that make these genes C_4_-suitable are not yet known and will be identified only through an exhaustive description of the expression patterns and catalytic properties of all members of several gene families. None of the gene lineages in the C_3_ ancestors were pre-optimized for the C_4_ pathway. Their expression levels and their diurnal regulation had to be altered during C_4_ evolution. It is also known that, in several cases, their catalytic properties have been optimized through key amino acid changes. Some gene lineages were, however, very likely closer to the requirements for C_4_ photosynthesis, and their presence in grass genomes would therefore have increased the evolvability of the C_4_ trait itself. Different suitability of the members of gene families for recruitment into novel traits also means that the evolutionary loss of some gene duplicates might, in the long term, limit future evolutionary trajectories. Orthologs of C_4_-specific forms are available in C_3_ grasses, with the notable exception of the PEPC gene lineage recurrently recruited for the C_4_ pathway, which is absent from the rice genome (supplementary table S2, Supplementary Material online). However, similar genes might be absent from other large families, which despite similar growth forms and ecology lack C_4_ taxa, partially accounting for the restriction of C_4_ origins to some groups of plants.

## Supplementary Material

Supplementary tables S1 and S2 and figures S1–S4 are available at *Genome Biology and Evolution* online (http://www.gbe.oxfordjournals.org/).

Supplementary Data
