# Supplementary material for: Parallel Recruitment of Multiple Genes into C4 Photosynthesis
Source: Genome Biol Evol. 2013 Oct 31;5(11):2174–87. doi: 10.1093/gbe/evt168 (PMC3845648; doi:10.1093/gbe/evt168)
Supplement: Supplementary Data [file supp_5_11_2174__index.html]

Parallel recruitment of multiple genes into C4 photosynthesis — Parallel Recruitment of Multiple Genes into C4 Photosynthesis — Supplementary Data 

# Parallel Recruitment of Multiple Genes into C4 Photosynthesis

## Supplementary Data

files

**Files in this Data Supplement:**

- Supplementary Data - pdf file
- Supplementary Data - xls file
