# Supplementary material for: Parallel Recruitment of Multiple Genes into C4 Photosynthesis
Source: Genome Biol Evol. 2013 Oct 31;5(11):2174–87. doi: 10.1093/gbe/evt168 (PMC3845648; doi:10.1093/gbe/evt168)
Supplement: Supplementary Data [file supp_evt168_Christin_SI.pdf]

Figure S1: Detailed schematic of the C_4 biochemical cycle.

The main enzymatic reactions of the C_4 cycles used by *Zea*, *Alloteropsis* and/or *Setaria* are indicated. Grey boxes represent enzymes. The malate shuttle is represented by red arrows and the aspartate shuttle by green arrows. Abbreviations as Fig. 1, plus: AK, adenylate kinase; PPa, inorganic pyrophasphatase**.**

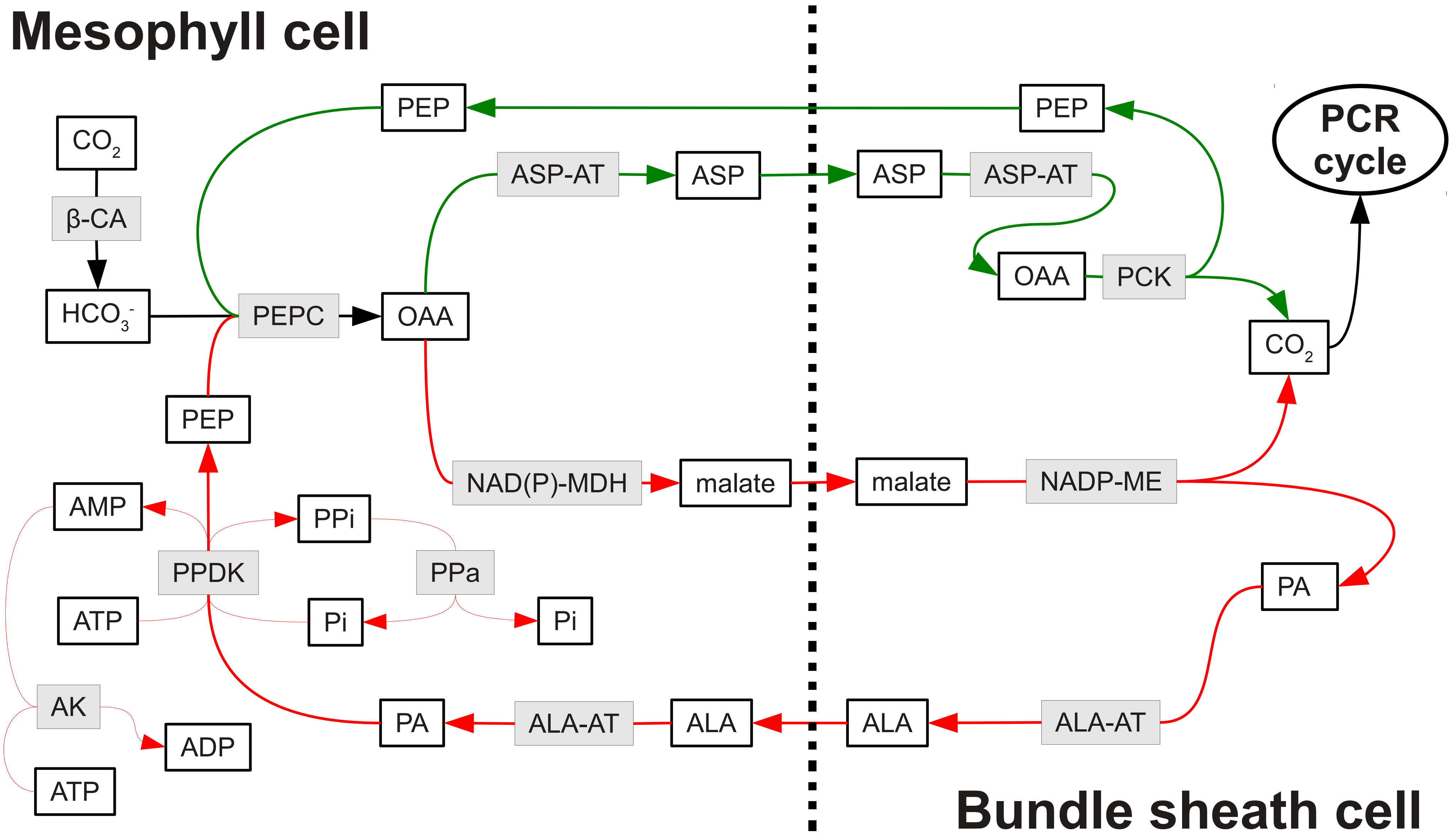

Mesophyll cell
PEP
PEP
PCR cycle
CO2
ASP-AT
ASP
ASP
ASP-AT
β-CA
OAA
PCK
HCO3-
PEPC
OAA
CO2
PEP
NAD(P)-MDH
malate
malate
NADP-ME
AMP
PPi
PPDK
PPa
PA
ATP
Pi
Pi
AK
ADP
PA
ALA-AT
ALA
ALA
ALA-AT
ATP
Bundle sheath cell

Figure S2: Diurnal regulation of putative transcription factors.

For seven transcription factors identified as candidates for a C_4 function, the normalized transcript abundance is indicated over the course of a day, with time shown in hours after dawn. Values are comparable within each panel but not among panels. For each sample point, standard errors were calculated from three replicates. Values measured in the C_3 *Alloteropsis* are in blue and those measured in the C_4 *Alloteropsis* are in red. The grey bar at the bottom represents the light period and the black bar the dark period.

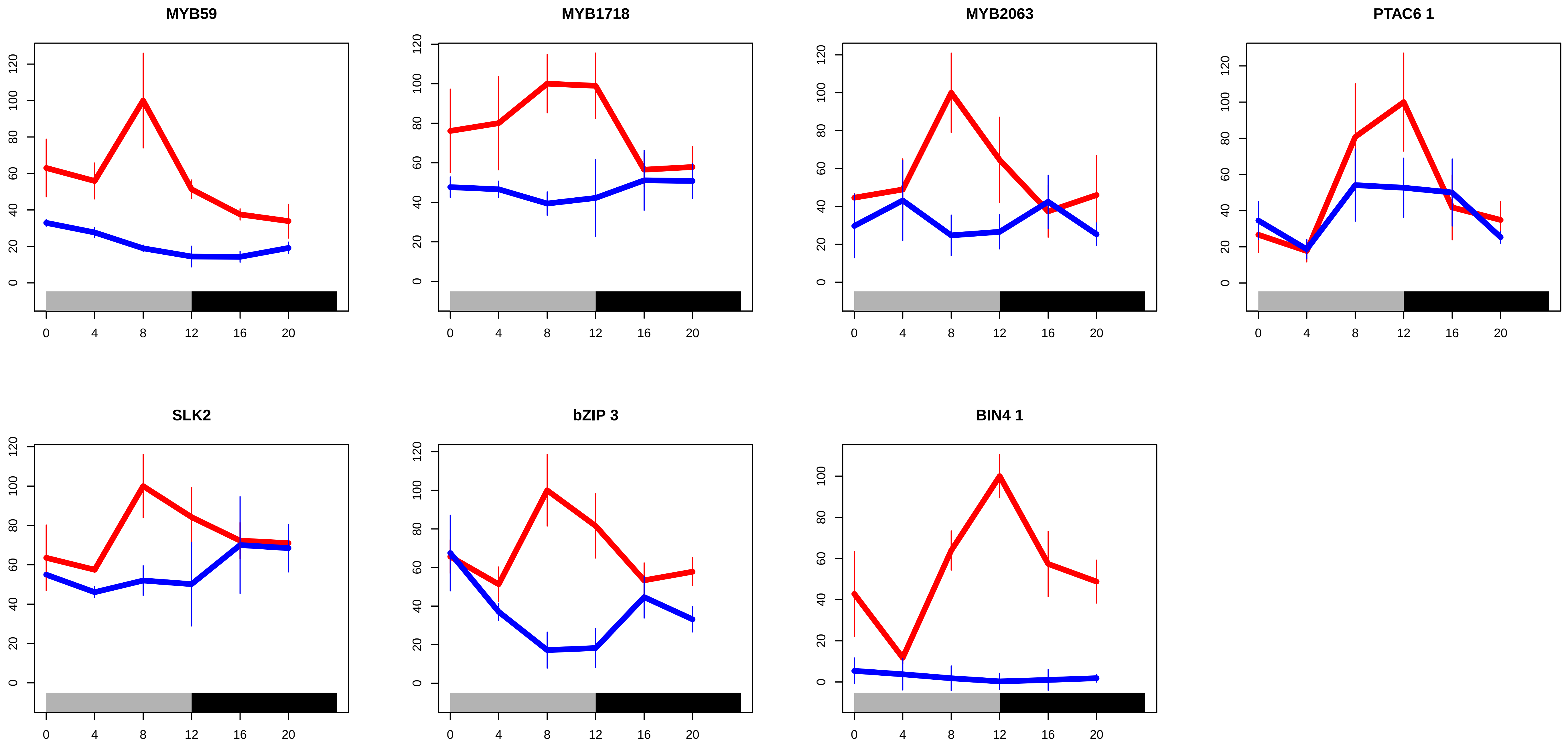
MYB59
MYB1718
MYB2063
PTAC6 1
SLK2
bZIP 3
BIN4 1
0
20
40
60
80
100
120
0
4
8
12
16
20

Figure S3: Phylogenetic trees inferred for C_4-related gene families.

For each C_4-related enzyme, the phylogenetic tree obtained under maximum likelihood is shown, with bootstrap support values indicated near branches. The grass gene lineages are delimited on the right and the genes predicted to lead to a chloroplast-specific expression are indicated with black circles. Asterisks indicate genes for which a system of dual promoters has been reported, one of which generates a protein targeted to the chloroplast and the other one a cytosolic protein. For each gene lineage, barplots on the right are proportional to the rpkm in different species (Ass = C_4 *Alloteropsis*; Ase = C_3 *Alloteropsis*; Si = *Setaria*; Zm = *Zea*), different conditions for *Alloteropsis* (black = day; grey = night) and different stages of development for *Setaria* and *Zea* (from left to right, A = base of the leaf; B = transitional; C = maturing; D = mature). When a putative C_4 form is identified, the rpkm values used for the identification are highlighted in red. The exact rpkm values for each species and each gene lineage are available in Table S2.

Adenylate kinase protein (AK)

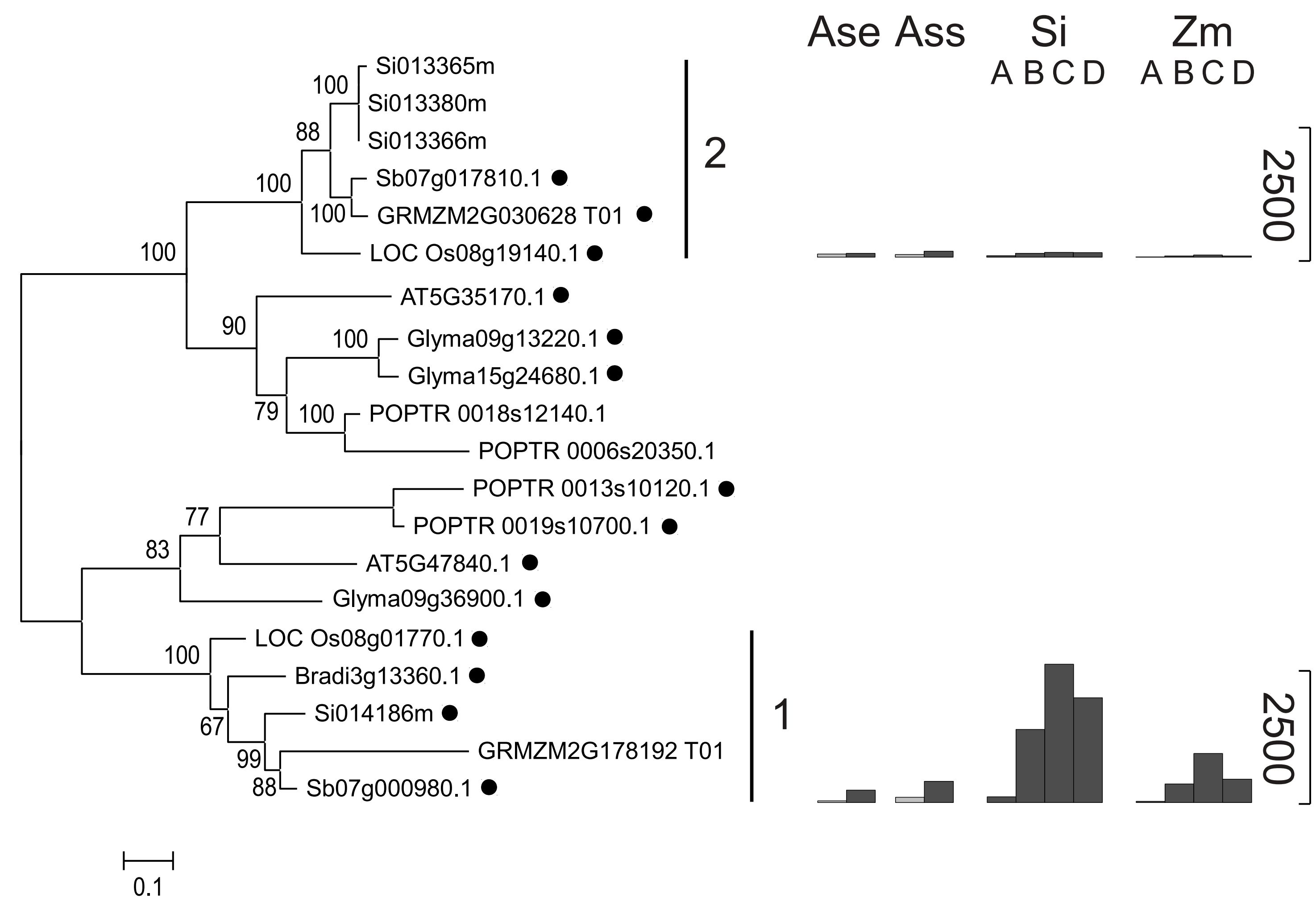

Alanine aminotransferase (ALA-AT)

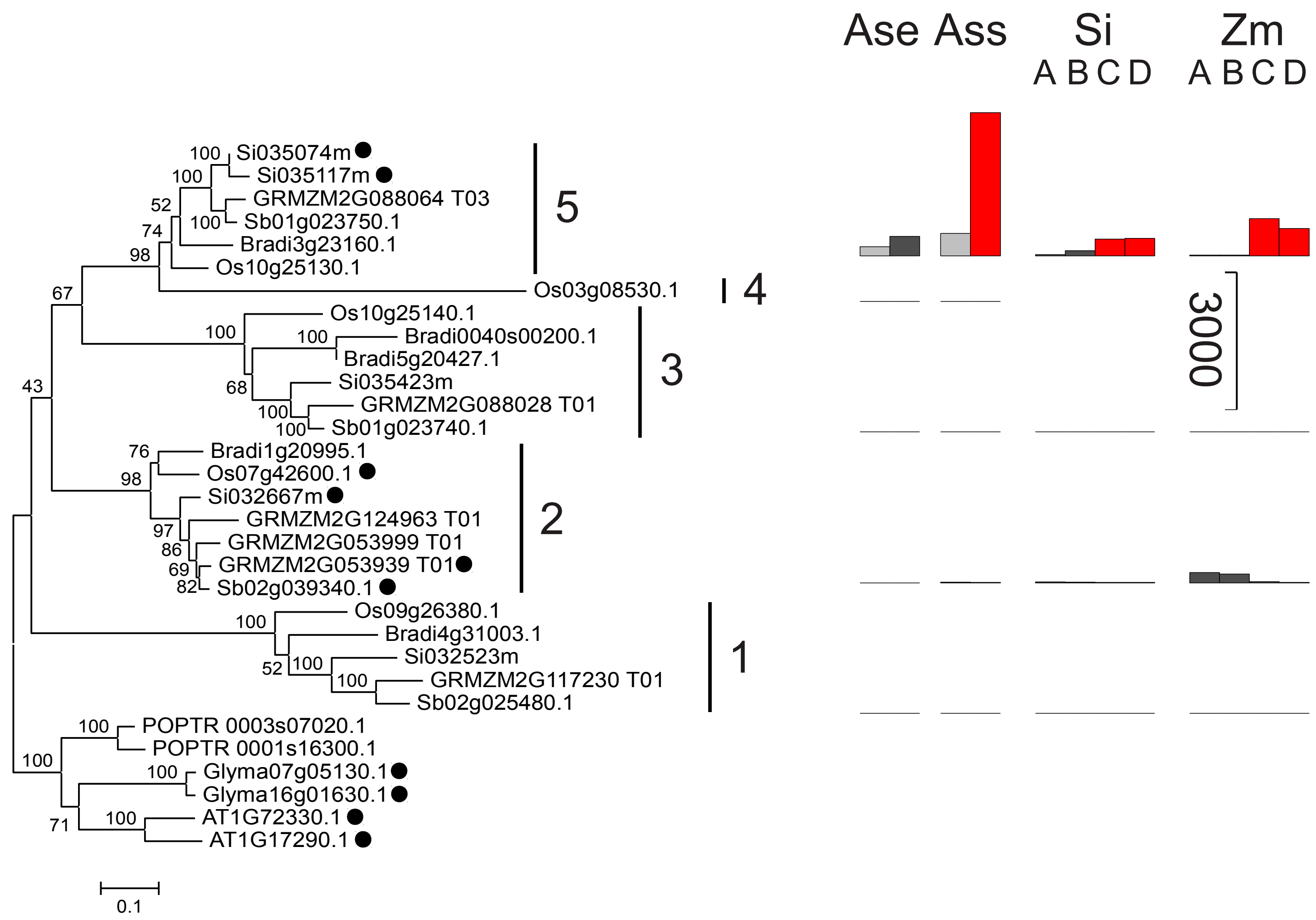

Aspartate aminotransferase (ASP-AT)

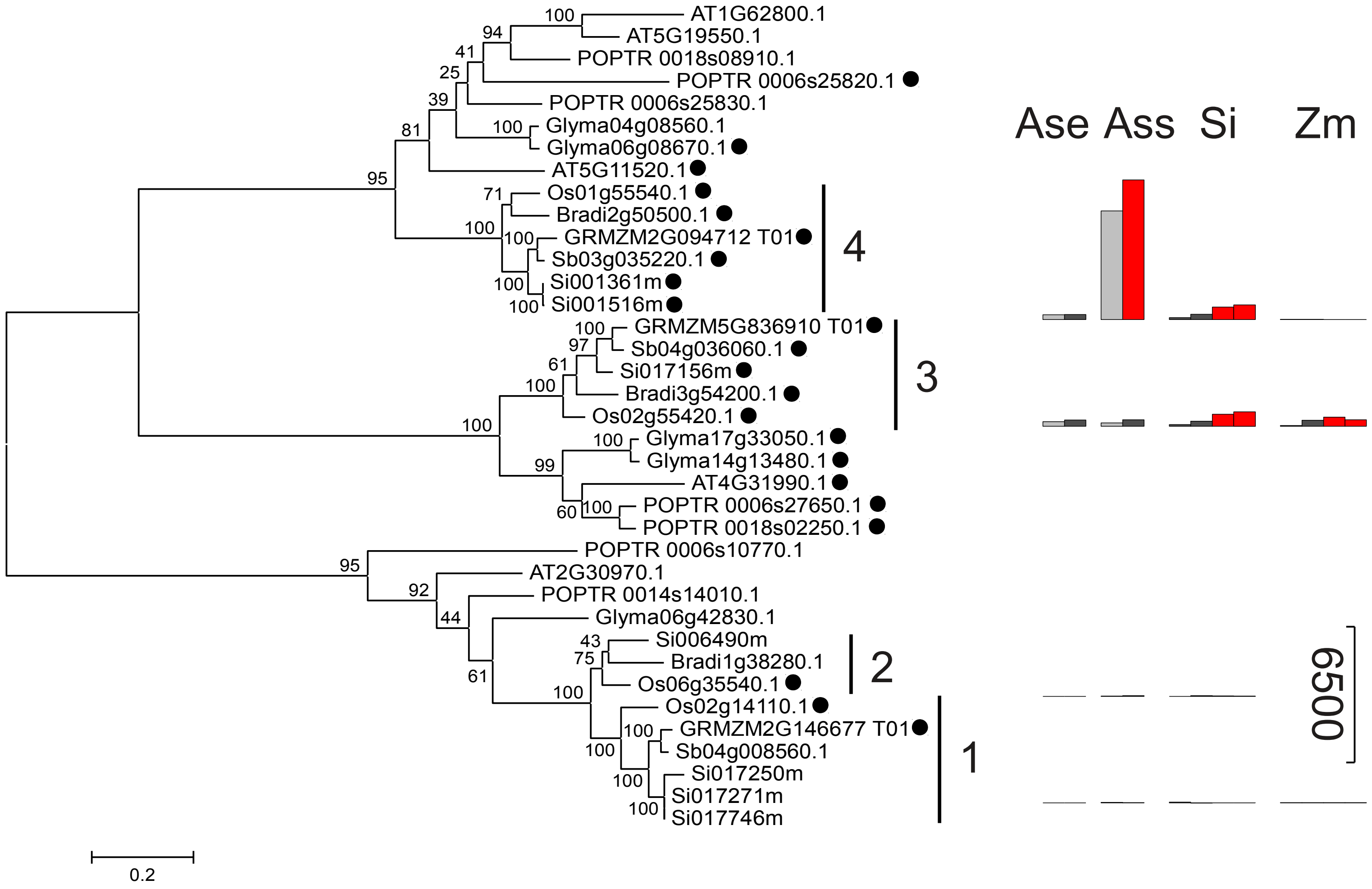

Beta carbonic anhydrase (BETA-CA)

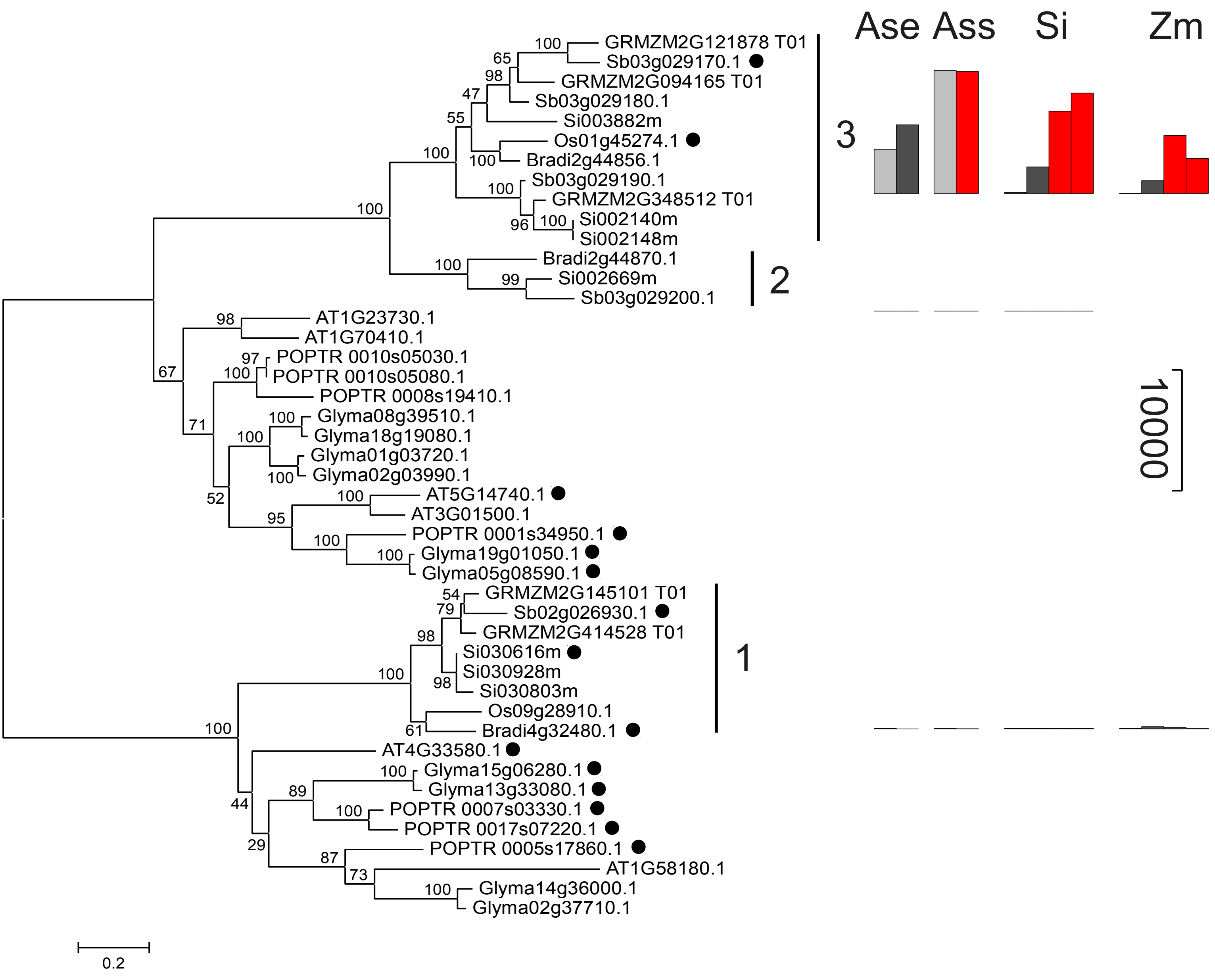

NAD-malic enzyme (NAD-ME)

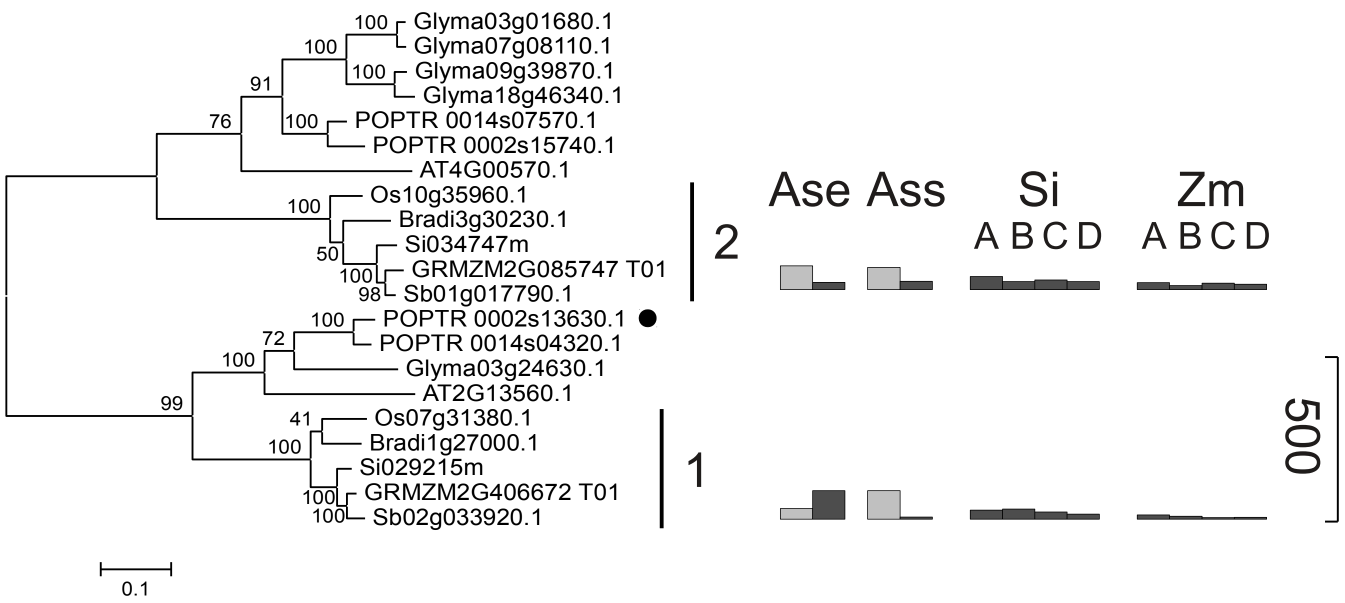

NADP-malic enzyme (NADP-ME)

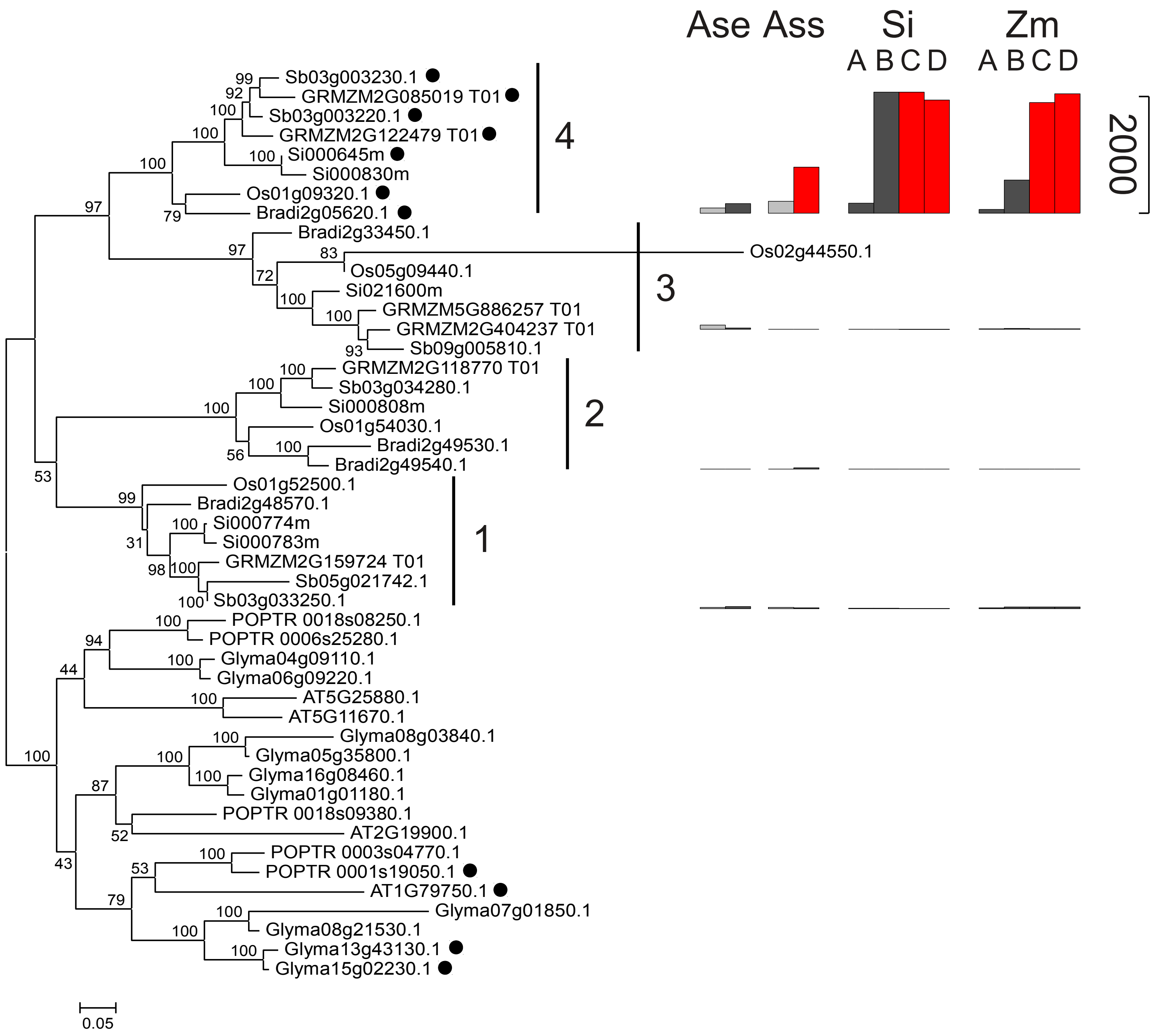

NAD-malate dehydrogenase (NAD-MDH)

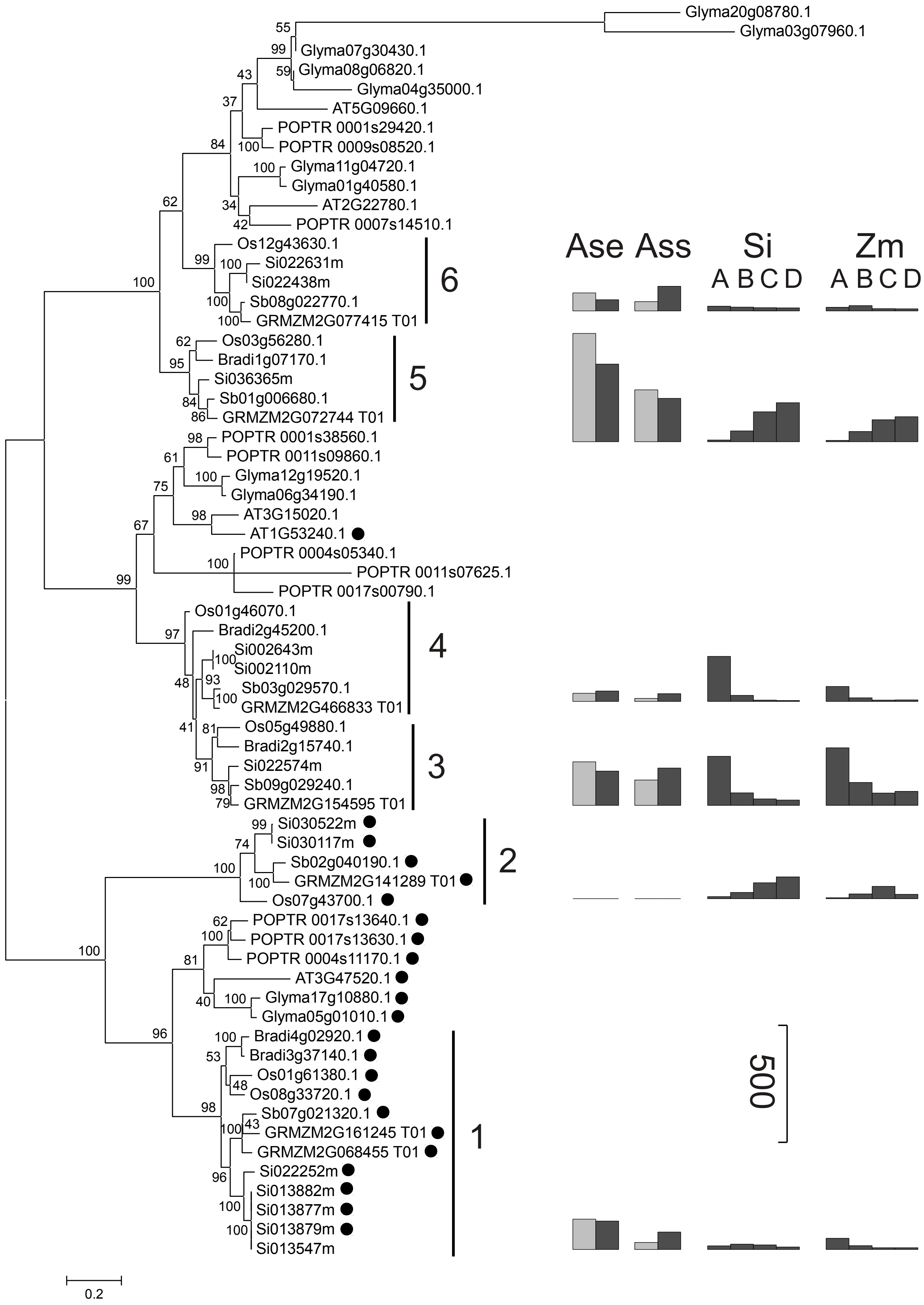

NAD(P)-malate dehydrogenase (NAD(P)-MDH)

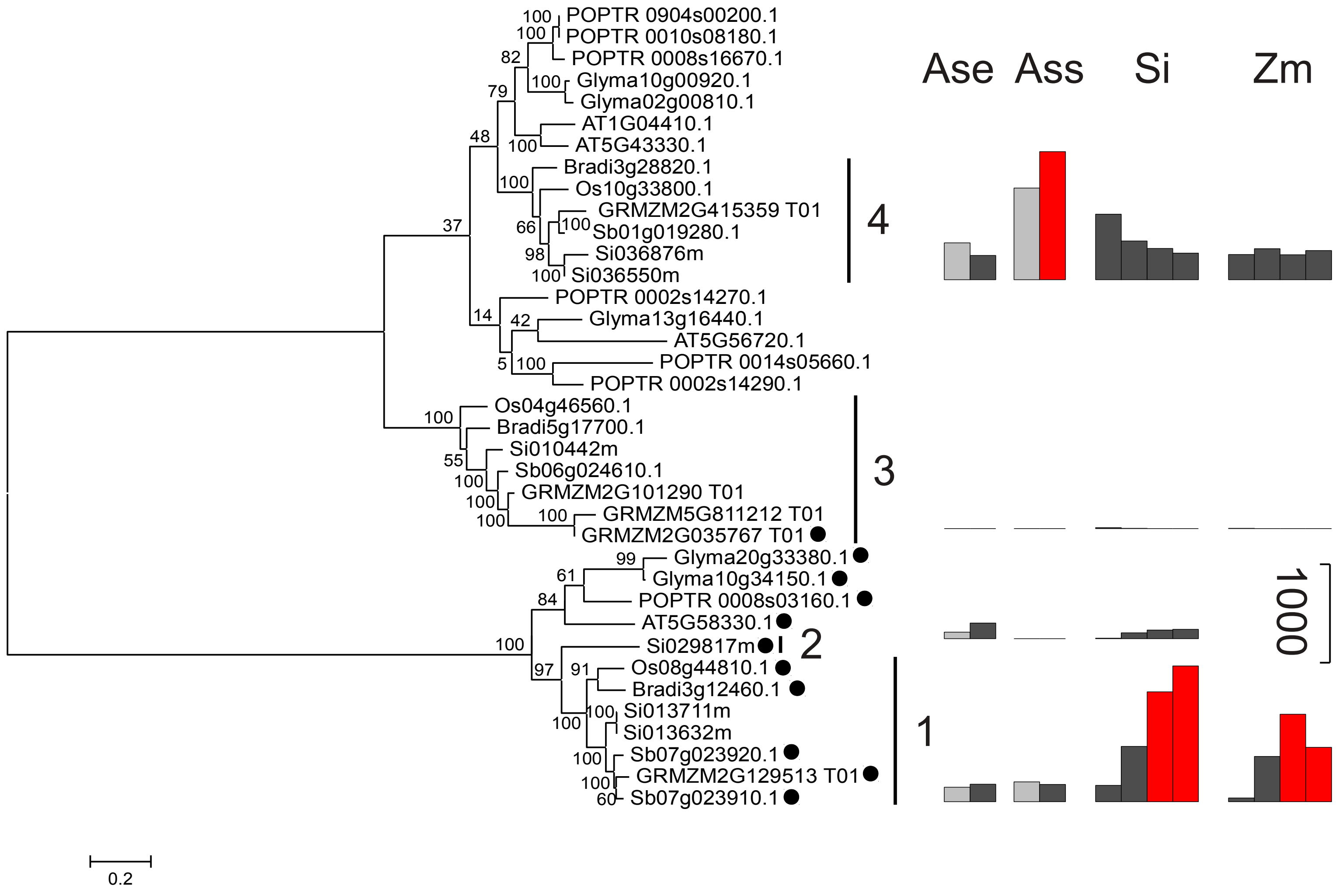

Phosphoenolpyruvate carboxykinase (PCK)

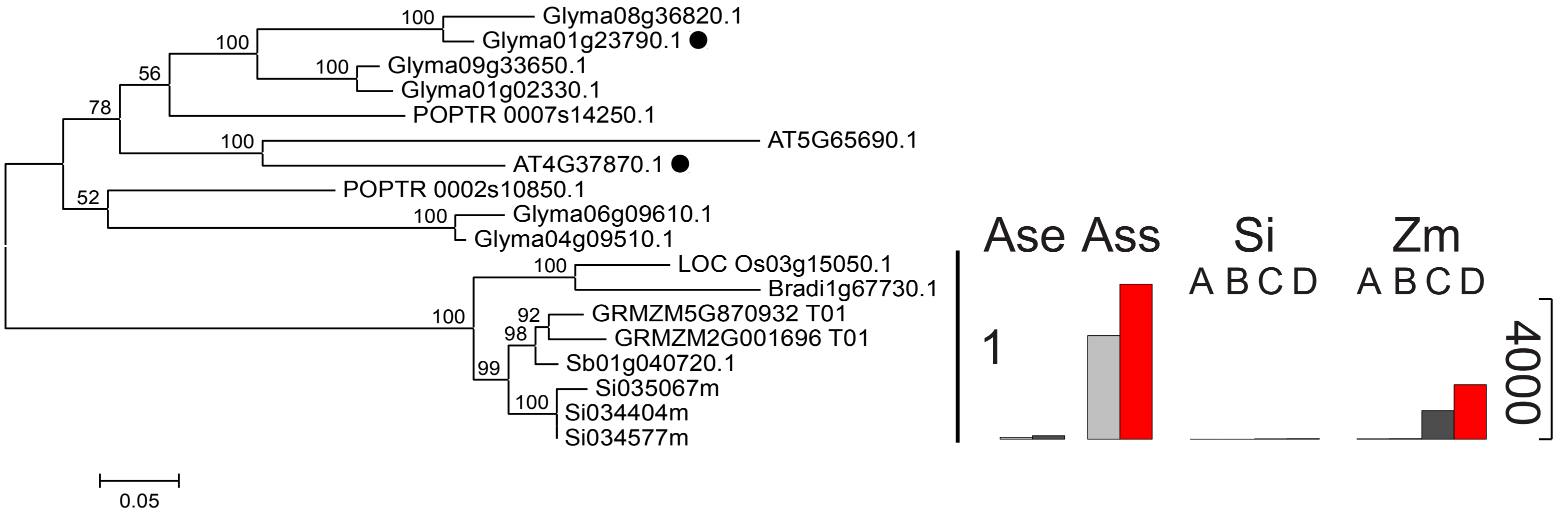

Phosphoenolpyruvate carboxylase (PEPC)

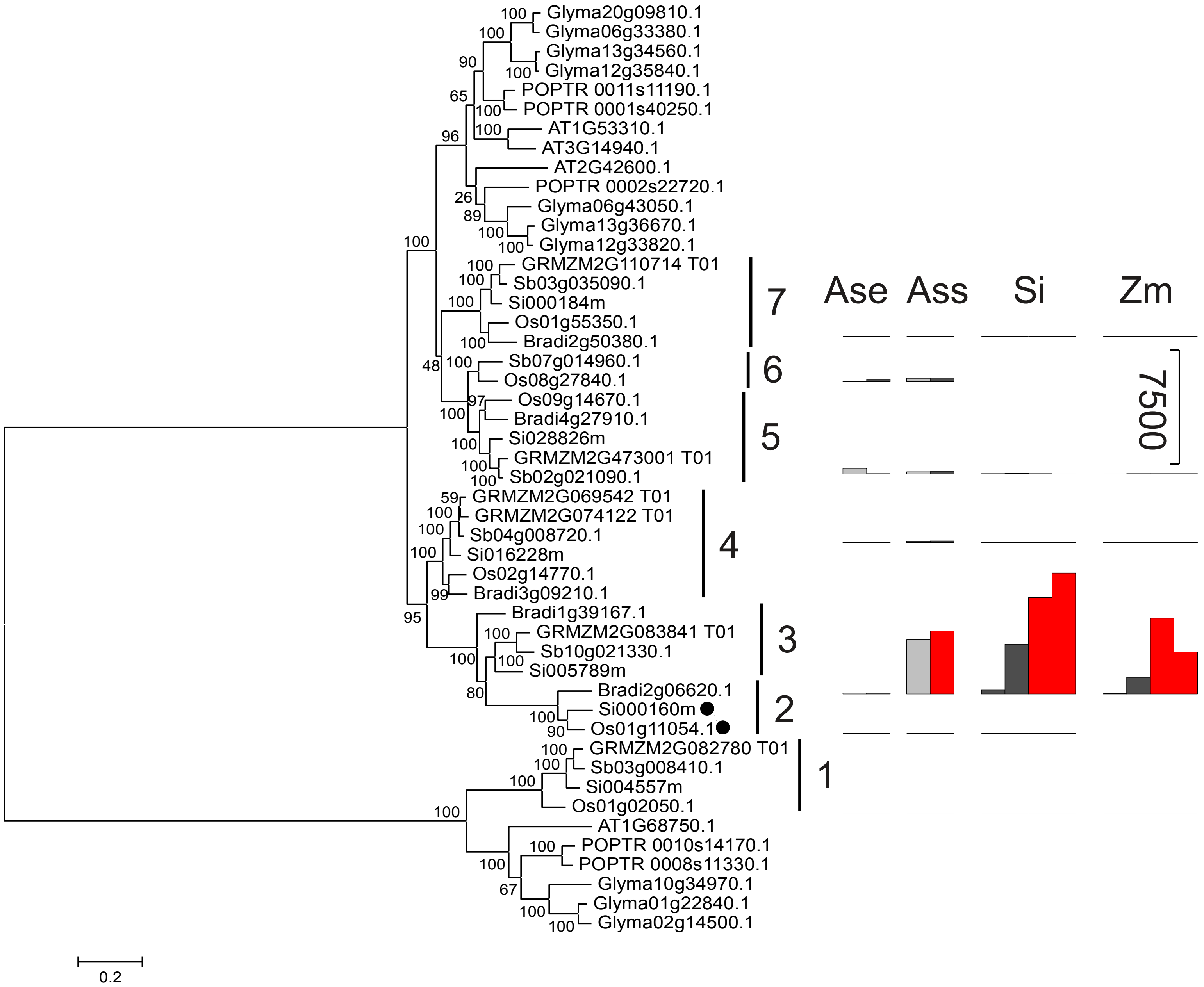

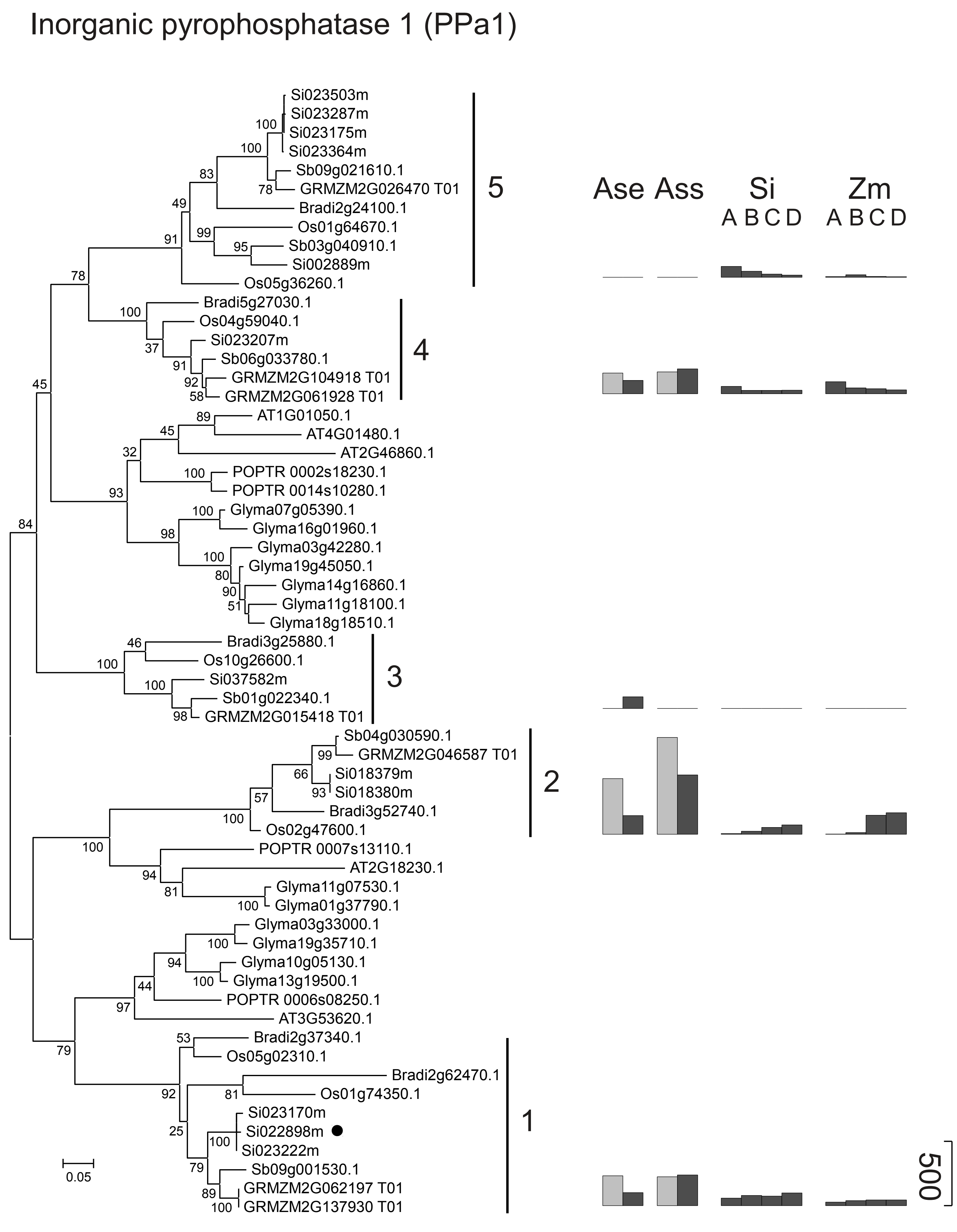

Inorganic pyrophosphatase 1 (PPa1)
Si023503m
Si023287m
Si023175m
Si023364m
Sb09g021610.1
GRMZM2G026470 T01
Bradi2g24100.1
Os01g64670.1
Sb03g040910.1
Si002889m
Os05g36260.1
Bradi5g27030.1
Os04g59040.1
Si023207m
Sb06g033780.1
GRMZM2G104918 T01
GRMZM2G061928 T01
AT1G01050.1
AT4G01480.1
AT2G46860.1
POPTR 0002s18230.1
POPTR 0014s10280.1
Glyma07g05390.1
Glyma16g01960.1
Glyma03g42280.1
Glyma19g45050.1
Glyma14g16860.1
Glyma11g18100.1
Glyma18g18510.1
Bradi3g25880.1
Os10g26600.1
Si037582m
Sb01g022340.1
GRMZM2G015418 T01
Sb04g030590.1
GRMZM2G046587 T01
Si018379m
Si018380m
Bradi3g52740.1
Os02g47600.1
POPTR 0007s13110.1
AT2G18230.1
Glyma11g07530.1
Glyma01g37790.1
Glyma03g33000.1
Glyma19g35710.1
Glyma10g05130.1
Glyma13g19500.1
POPTR 0006s08250.1
AT3G53620.1
Bradi2g37340.1
Os05g02310.1
Bradi2g62470.1
Os01g74350.1
Si023170m
Si022898m
Si023222m
Sb09g001530.1
GRMZM2G062197 T01
GRMZM2G137930 T01
5
4
3
2
1
Ase Ass
Si
A B C D
Zm
A B C D
0.05
500

Inorganic pyrophosphatase 2 (PPa2)

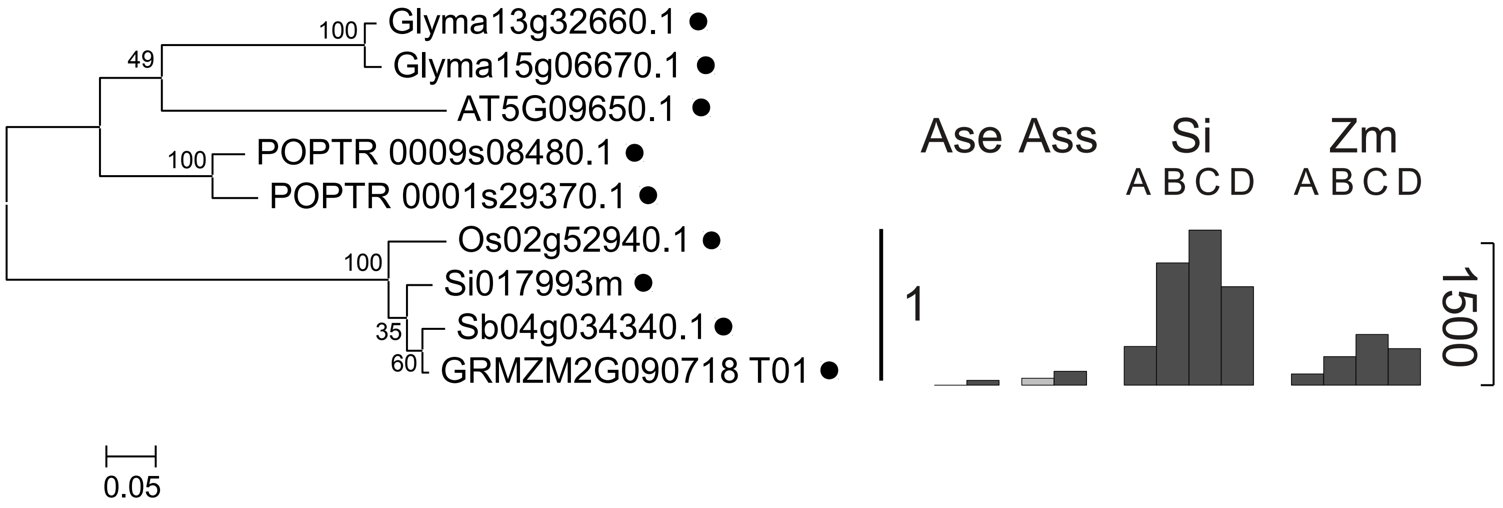

Pyruvate, phosphate dikinase (PPDK)

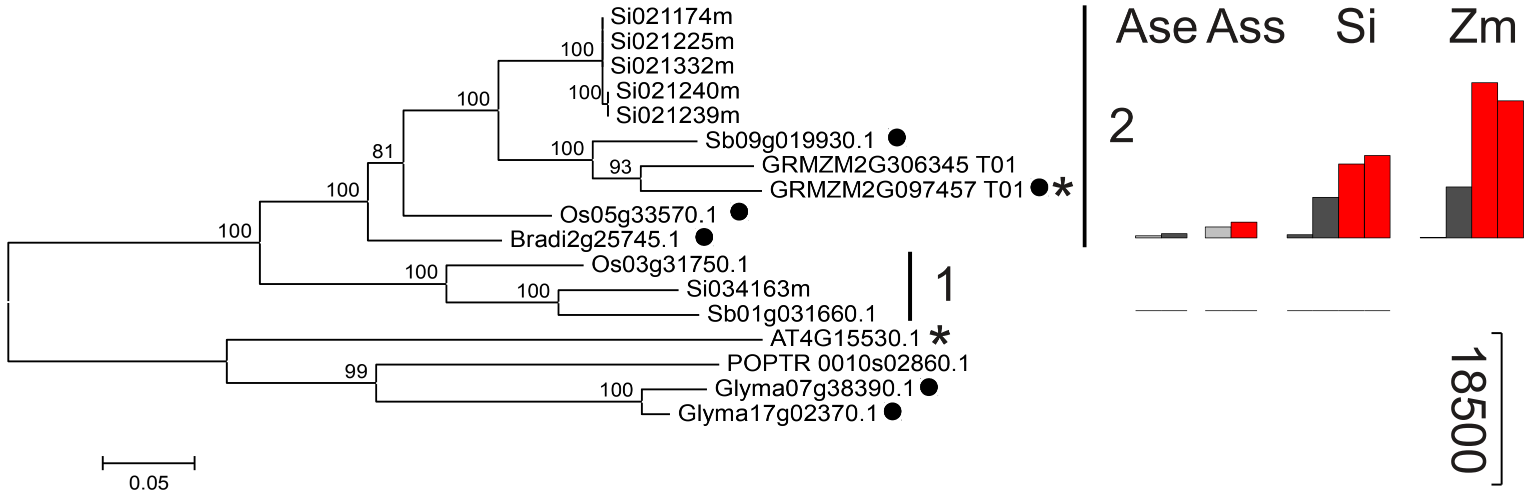

Pyruvate, phosphate dikinase regulatory protein (PPDK-RP)

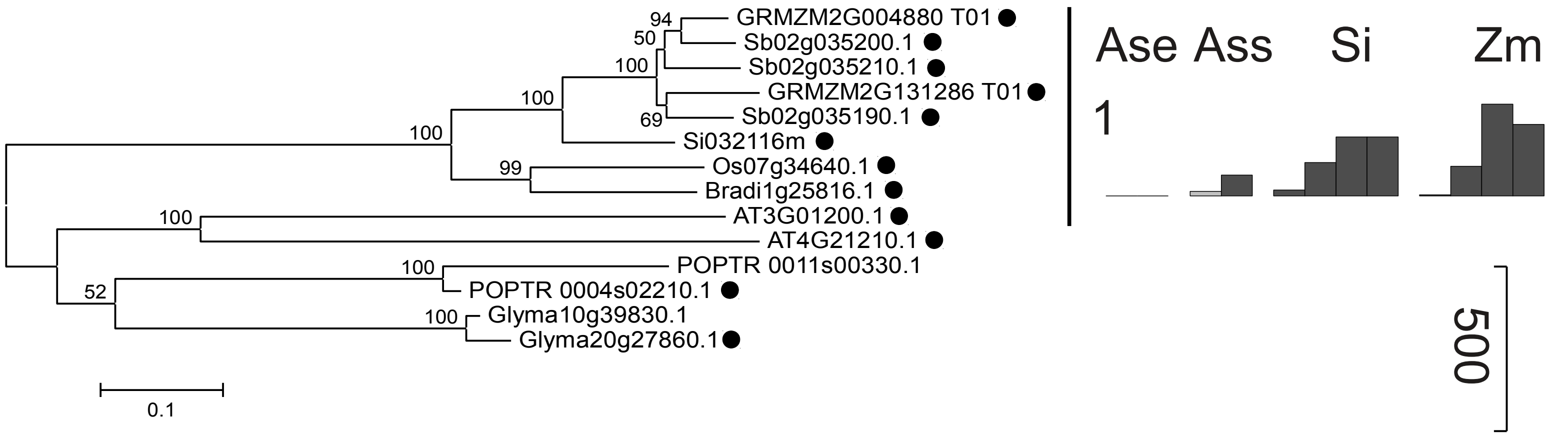

Sodium bile acid symporter-like protein (BASS2)

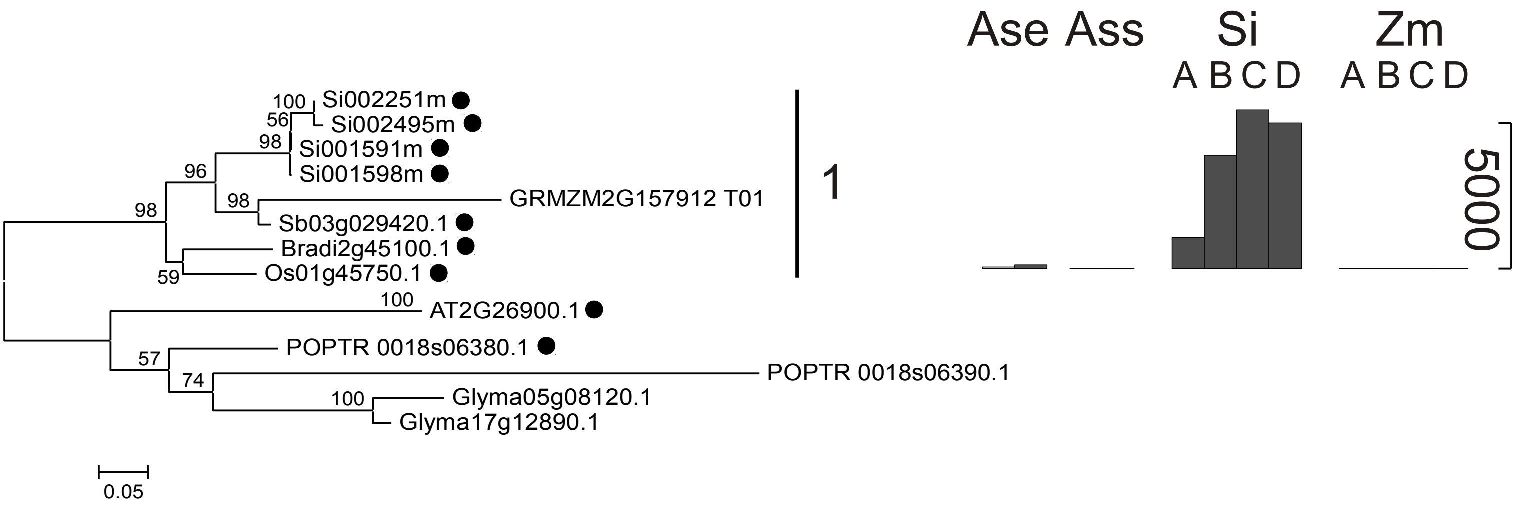

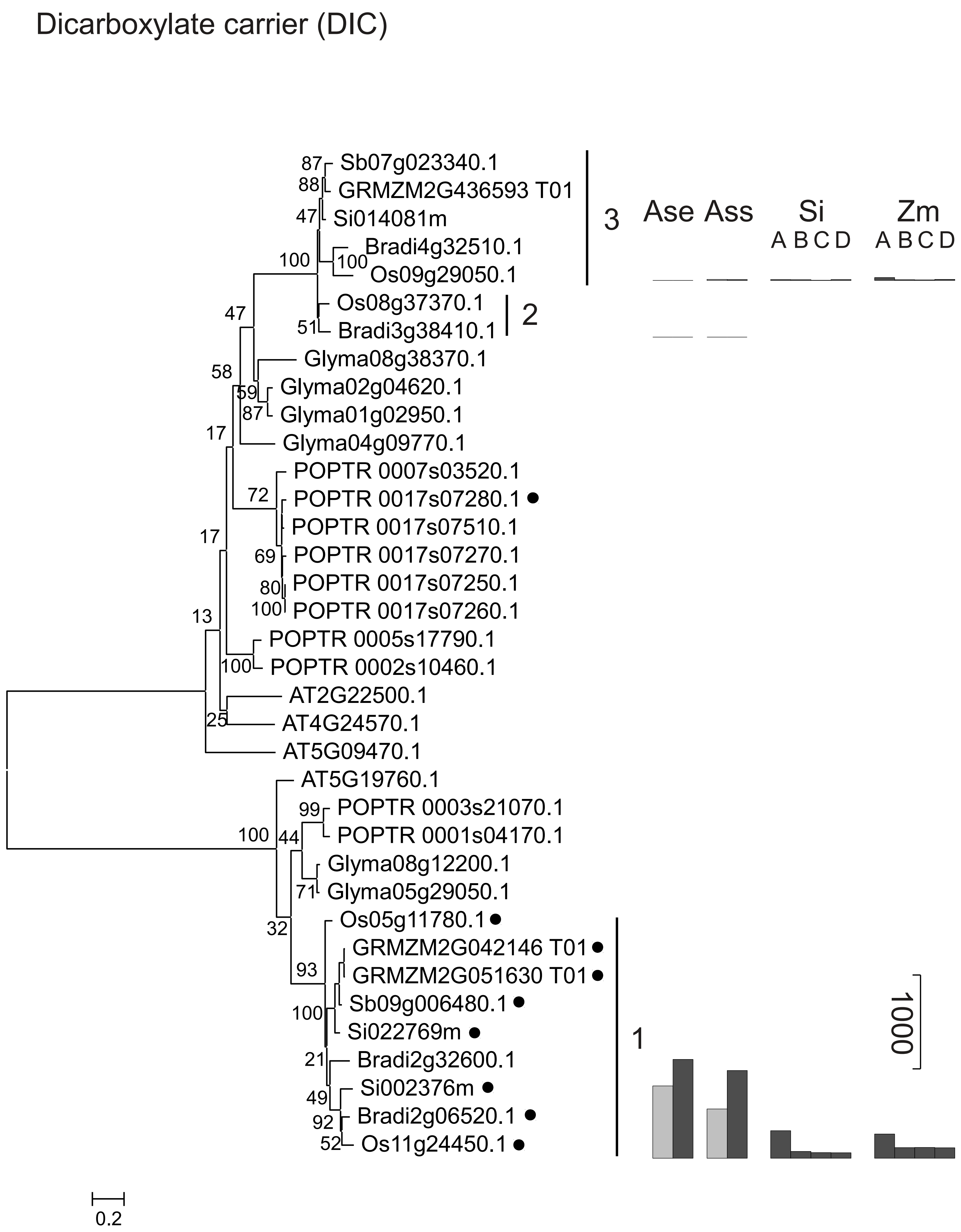
Dicarboxylate carrier (DIC)
Sb07g023340.1
GRMZM2G436593 T01
Si014081m
Bradi4g32510.1
Os09g29050.1
Os08g37370.1
Bradi3g38410.1
Glyma08g38370.1
Glyma02g04620.1
Glyma01g02950.1
Glyma04g09770.1
POPTR 0007s03520.1
POPTR 0017s07280.1
POPTR 0017s07510.1
POPTR 0017s07270.1
POPTR 0017s07250.1
POPTR 0017s07260.1
POPTR 0005s17790.1
POPTR 0002s10460.1
AT2G22500.1
AT4G24570.1
AT5G09470.1
AT5G19760.1
POPTR 0003s21070.1
POPTR 0001s04170.1
Glyma08g12200.1
Glyma05g29050.1
Os05g11780.1
GRMZM2G042146 T01
GRMZM2G051630 T01
Sb09g006480.1
Si022769m
Bradi2g32600.1
Si002376m
Bradi2g06520.1
Os11g24450.1
3
2
1
Ase Ass
Si
A B C D
Zm
A B C D
1000
0.2

Dicarboxylate transporter (DIT)

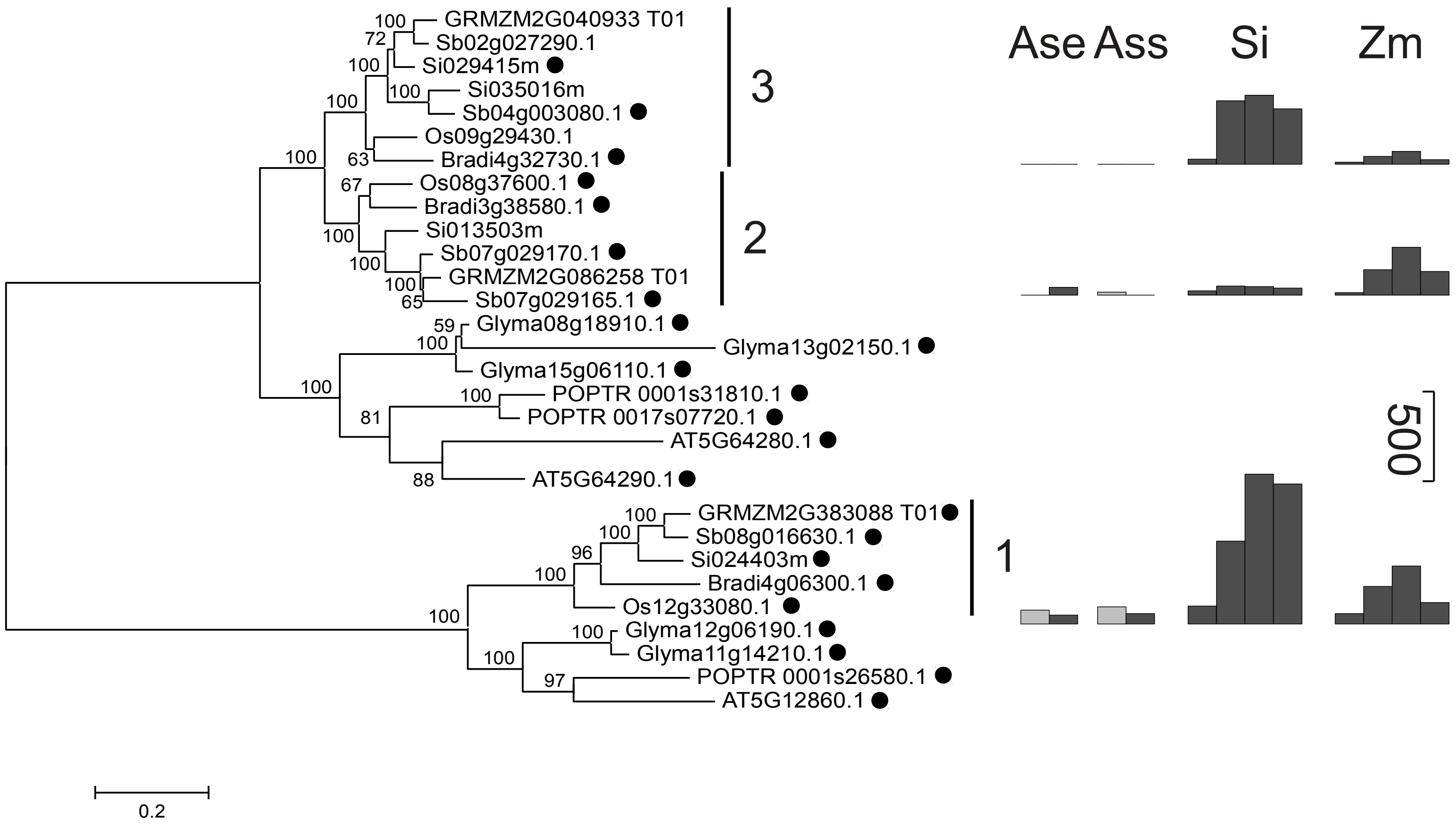

Phosphoenolpyruvate/phosphate translocator (PPT)/ Triosephosphate phosphate translocator (TPT)

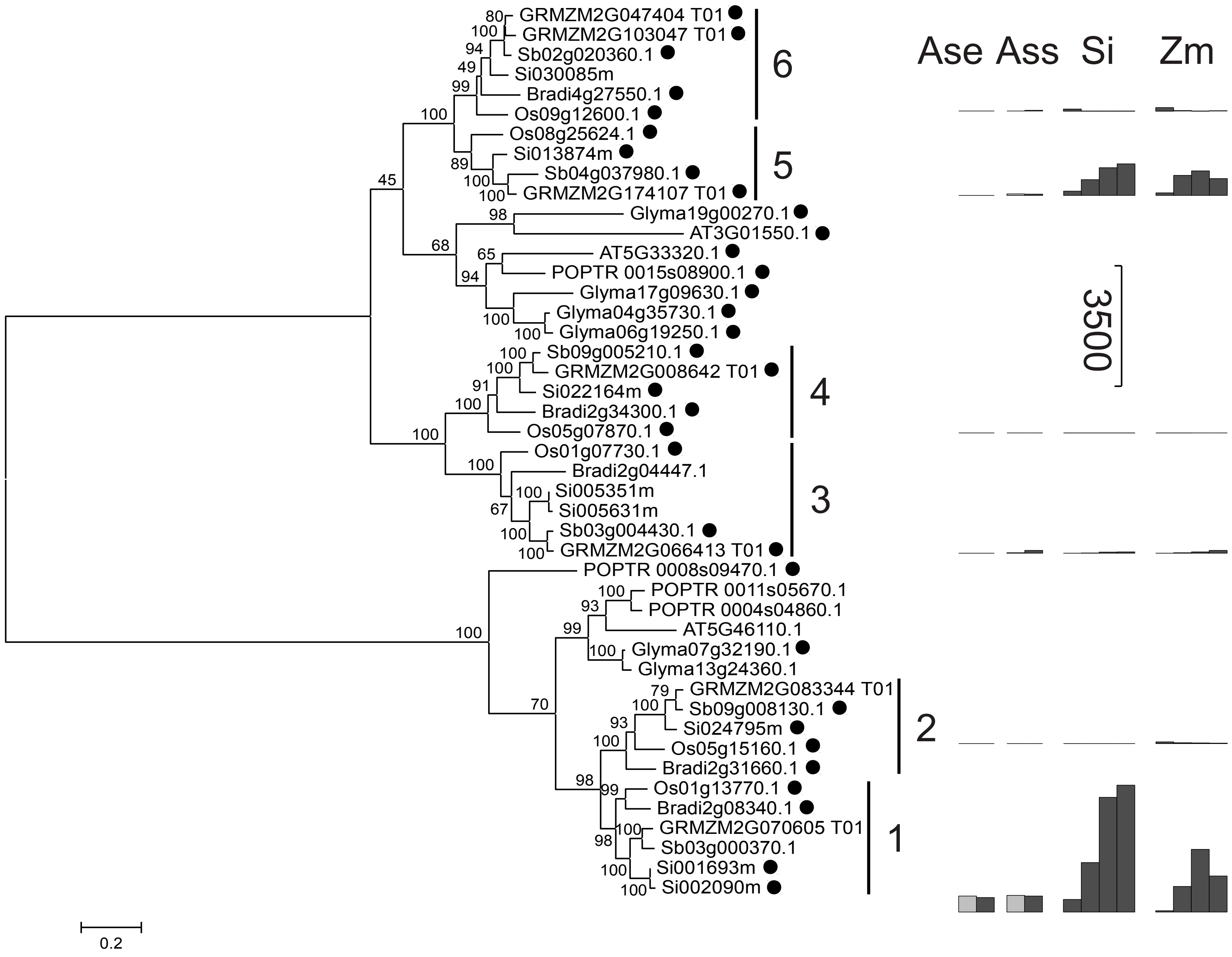

Figure S4: Phylogenetic trees inferred for transcription factors with a putative C_4 function. For each transcription factor, the phylogenetic tree obtained under maximum likelihood is shown, with bootstrap support values indicated near branches. The grass gene lineages are delimited on the right and the genes predicted to lead to a chloroplast-specific expression are indicated with black circles. For each gene lineage, barplots on the right are proportional to the rpkm in different species (Ass = C_4 *Alloteropsis*; Ase = C_3 *Alloteropsis*; Si = *Setaria*; Zm = *Zea*), different conditions for *Alloteropsis* (black = day; grey = night) and different stages of development for *Setaria* and *Zea* (A = base of the leaf; B = transitional; C = maturing; D = mature). The exact rpkm values for each species and each gene lineage are available in Table S2.

DNA-binding protein BIN4 (BIN4)

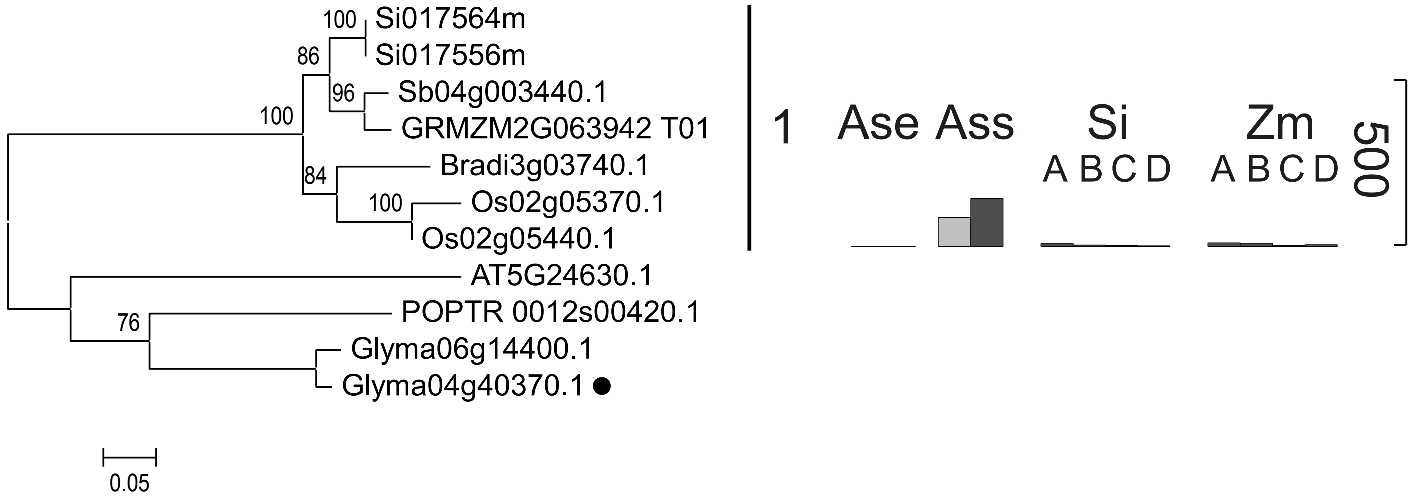

Basic leucine zipper transcription factor (bZIP)

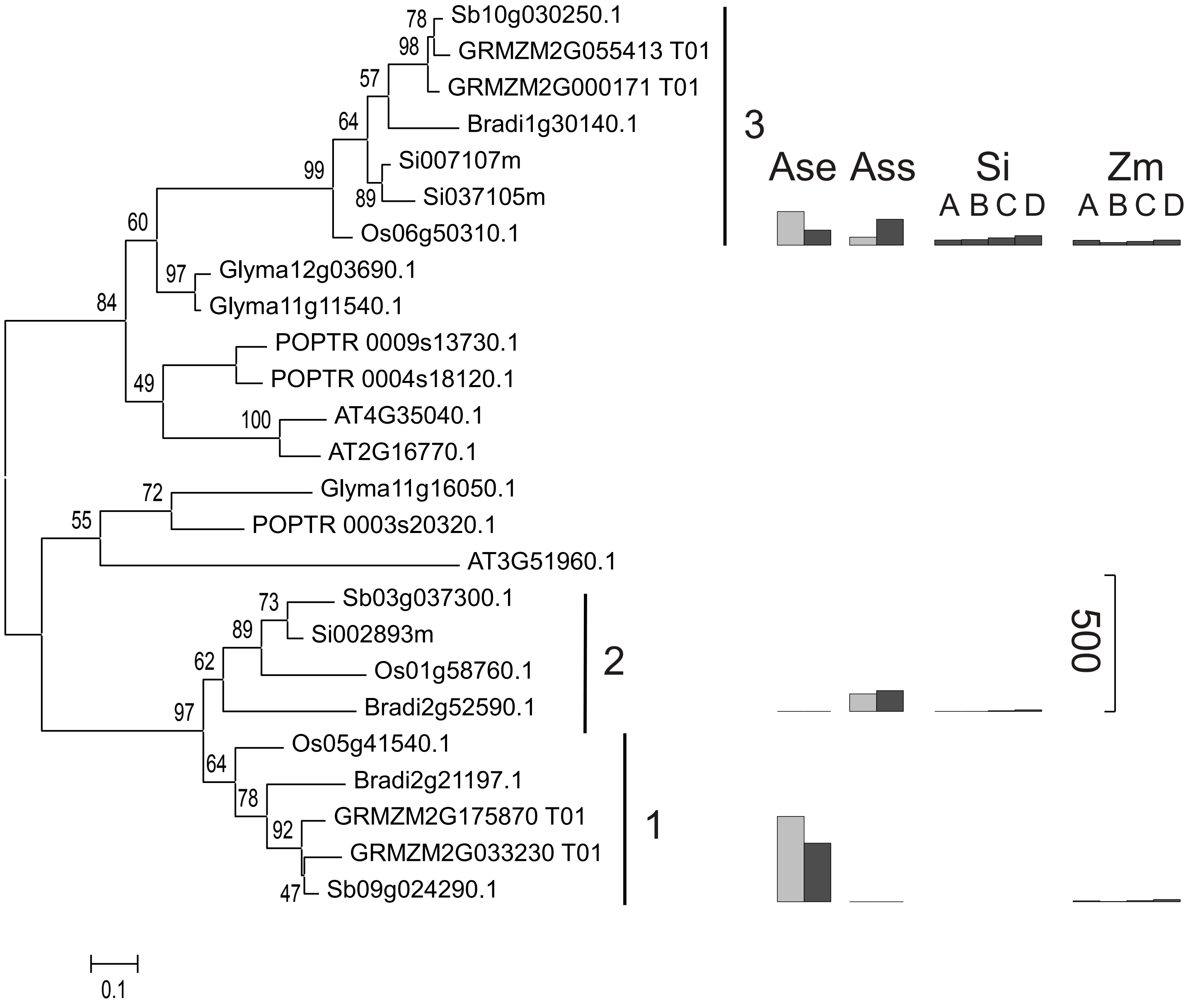

Plastid transcriptionally active 6 (PTAC6)

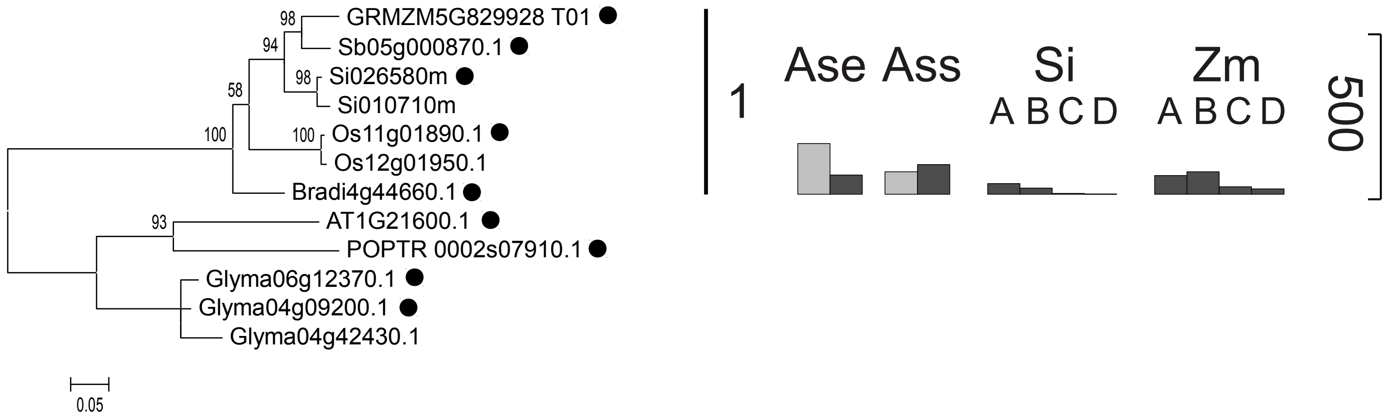

Table S1: List of primers used for semi-quantitative PCR.

Gene	Lineage	Forward	Reverse
β-CA	3	CGCCGACTCCCGTTGCTGCCCGTC	GGCCGCCGACCAGCTTGAGGGTCC
PEPC	3	GCAGCTTCCCGGCCAGCACGCCAT	CGCGTCGCGGTCGCGCTCGTACTC
PPCK	NA	TACTTGATGCTCTCCGGGACCGTG	TTCTCCTCCTCGACGAACGCCATT
ASP-AT	4	TTCCCCACCATGGCGTCTCAGG	GGATTGCAGGACTGTCAGCACCAA
PCK	1	GTACGCGGGAGAGATGAAGAAGG	CTGATGAACTGGTACATGGTCTG
PPDK	2	GCGGCGACCGCTTCGCCTACGACT	CACAGCAGTGCCTCGCAGGCCGGT
AK	1	CCCATGGCTTCCTCCATGGCCGC	CACCAAACCATATTTGGCCTTGATGAGC
PPa2	1	ACTTCGTCGTCGAGATCCCCAAGG	ATTTGCAGAAGTCGGGTCCTCCCA
DIC	1	CTGCGAGGCTAGGAGGAGGAGGAG	TCCAAGACGAGCGGTCGTGTACGTG
MYB59	NA	ATGCAACTGGTATGCACTGTCCGC	TCTCATTGTCTGTGCGCCCTGGTA
MYB1718	NA	GTCATGCCAATGCCAGGAGCTGAT	TTGAGGCAACAACCTCCTCTCCCA
MYB2063	NA	GCCCAGCTTGGTGGACCAGATAGA	AGCCGCTTCTGAACCTCCATTTGC
PTAC6	1	CTTTATCCGAAAGGTGCCCGACCC	CGCAAATGCTCAACTGGTGGCTCA
SLK2	NA	TCAGCAGCTGGTCCATGGTCAGAA	CCTAACTGCATTCCAGGCTGTGCC
bZIP	3	CAGTCCATGACCATTCGCACACCC	CCGCGTGATTCTGCAGCTTCTTCA
BIN4	1	AGGCACTCGGCTCCAAAGAAGGAT	TCCCAACAGCACCAATATCTCCGC
UBQ10	NA	CCTCACTGGCAAGACCATCACCCT	GAAGATCTGCATCCCACCCCTCAG

Table S2: Gene families and transcription levels

Gene lineages are listed for each of the C_4-related enzymes and selected transcription factors, with the gene identification for the five complete grass genomes. The expression levels are indicated in rpkm for the day and night samples of the C_4 *Alloteropsis* (Ass) as well as the C_3 (Ase), for the four samples of *Setaria* (Si; A = 0-1 cm above the leaf-four ligule, B = 1-0 cm below the leaf-three ligule, C = 2-3 cm above the leaf-three ligule, D = 2-1 cm below the leaf-four tip) and four samples of *Zea* (Zm; A = 1cm above the leaf three ligule, B = 1cm below the leaf two ligule, C = 4cm above the leaf two ligule, D = 1cm below the leaf three tip). For each multigene family, the putative C_4-specific form is highlighted in green. Additional putative C_4-specific lineages that were not included in the tally of convergent recruitment because the evidence was not judged conclusive in all cases (see text) are highlighted in blue.
